# ﻿Characterization of 11 complete mitochondrial genomes in Nudibranchia (Mollusca, Gastropoda)

**DOI:** 10.3897/zookeys.1244.139617

**Published:** 2025-07-04

**Authors:** Wanying Li, Fengping Li, Mingjie Liu, Aimin Wang, Chunsheng Liu, Yi Yang

**Affiliations:** 1 School of Marine Biology and Fisheries, Hainan University, Haikou 570228, China Hainan University Haikou China; 2 Sanya Nanfan Research Institute, Hainan University, Sanya 572025, China Sanya Nanfan Research Institute, Hainan University Sanya China

**Keywords:** Heterobranchia, misidentifications, monophyly, phylogeny, systematics

## Abstract

Nudibranchs, also known as sea slugs, represent a high species diversity within Heterobranchia (Mollusca, Gastropoda); however, the systematics within this order remain controversial. The mitochondrial genome has been widely used in the phylogenetic reconstruction of mollusks, but the corresponding data on Nudibranchia are limited, hindering understanding of the phylogenetic relationships of this group. In the present study, the complete mitochondrial genomes of 11 species belonging to 8 families and 10 genera were newly sequenced and compared with the 64 previously published mitogenomes for analysis. All newly sequenced mitogenomes were double-stranded circular molecules ranging from 14,299 to 14,880 bp. A total of 37 genes, including 13 protein-coding genes (PCGs), two ribosomal RNA genes (rRNA), and 22 transfer RNA (tRNA) genes, were encoded among all species. Of these 37 genes, 24 are encoded on the heavy strand and 13 on the light strand. The mitogenomes showed variations in AT content, GC skew, and AT skew. The gene orders of Nudibranchia indicated that gene transpositions and inversion had occurred within *Hypselodoris*. Based on the nucleotide sequences of 13 PCGs and 2 rRNA genes, the phylogenetic relationships of Nudibranchia have been reconstructed at family level. The phylogenetic analyses confirmed that Nudibranchia can be divided into Doridina and Cladobranchia with high support. However, the monophyly of Chromodoridoidea within Doridina is rejected due to the isolated positions of Actinocyclidae and Cadlinidae. Within Cladobranchia, the non-monophyly of Fionoidea was revealed since the fionoid group Coryphellidae clusters with Aeolidioidea. Molecular evidence, along with morphological characteristics, indicates that misidentifications of nudibranch species has been frequent in public databases. The present study suggests that the systematics within Nudibranchia needs further revision.

## ﻿Introduction

As one of the largest groups of Heterobranchia (Mollusca, Gastropoda), the Nudibranchia represents more than 4,700 known species (Anderson 1995; Dean and Prinsep 2017), which belong to two major branches, the Cladobranchia and the Doridina (Bouchet et al. 2017). Nudibranchs, sometimes referred to as the “butterflies of the sea”, are widely distributed around the world but predominantly inhabiting in shallow tropical waters to a depth of 30 m (Wägele and Kolb 2005). The deep sea, however, still harbors numerous species that remain undiscovered to this day (e.g., Fahey and Gosliner 2000; Valdés 2008; Stout et al. 2011; Martynov 2013; Martynov and Korshunova 2015; Valdés et al. 2018). The intriguing defense mechanisms of nudibranchs have been a research focus in recent years. For instance, nudibranchs can sequester toxins from cnidarians and poriferans for self defense (e.g., Faulkner and Ghiselin 1983; Greenwood 2009). Nudibranchs are also highly tolerant of coastal environments, making them valuable as bioindicators (Ah-Shee-Tee et al. 2022; Garner and Oosthuizen 2023). Furthermore, nudibranchs can synthesize unpleasant or toxic compounds, providing new insights for discovery of chemical compounds with pharmaceutical relevance (Böhringer et al. 2017; Gomes et al. 2018). To better utilize these biological resources, a clear systematic framework is necessary.

The classification of Nudibranchia was begun by Cuvier (1817) who realized that nudibranchs were more related to snails with shells than to worms. The traditional classification of Nudibranchia was divided into four main taxa: the Doridoidea, Dendronotoidea, Arminoidea, and Aeolidioidea (Odhner 1934). However, the monophyly of Arminoidea was subsequently rejected (Wägele and Willan 2000). A morphological phylogeny recognized two major nudibranch groups based on the digestive system, the Anthobranchia (Doridina: Bathydoridoidea and Doridoidea) and the Cladobranchia (Dendronotoidea, Aeolidioidea, and Arminoidea) (Wägele and Willan 2000). The validity of the two major groups (Doridina and Cladobranchia) is still recognized (Bouchet et al. 2017), whereas the boundaries of several internal taxa were revised according to new phylogenetic analyses (Hallas and Gosliner 2015; Mahguib and Valdés 2015; Goodheart et al. 2017). The monophyly of Nudibranchia has been widely accepted based on both morphological and molecular evidence (Wägele 1998; Wollscheid and Wägele 1999; Zapata et al. 2014). Nevertheless, the internal phylogenetic relationships at higher levels within the Nudibranchia are less well understood, as previous molecular phylogenies were mostly reconstructed using one or several short markers and failed to arrive at well-supported topologies (Wollscheid-Lengeling et al. 2001; Pola and Gosliner 2010; Korshunova et al. 2020, 2021).

The typical mitochondrial genome of mollusks contains 13 protein-coding genes (PCGs), 22 transfer RNA genes (tRNAs), and two ribosomal RNA genes (rRNAs) (Sevigny et al. 2015; Do et al. 2022; Galià-Camps et al. 2024). Mitogenomic sequences have been proven to resolve phylogenetic relationships among various metazoans (Miya et al. 2001; Osigus et al. 2013; Li et al. 2015; Mikkelsen et al. 2018; Varney et al. 2021; Galià-Camps et al. 2024), providing higher resolution in phylogenetic analyses compared to single-gene or multi-gene fragments (Liu et al. 2022). Additionally, the complete mitochondrial genome contains structural information (e.g., genome organization, gene order rearrangements, synonymous and non-synonymous substitutions in PCGs, and RNA secondary structures), which may also provide phylogenetic signals (Milbury et al. 2010; Sun et al. 2018; Kong et al. 2020).

Although several nudibranch mitogenomes have been reported in previous studies (Sevigny et al. 2015; Lin et al. 2019; Kim et al. 2021; Do et al. 2022; Li et al. 2022a; Liu et al. 2023; Mizobata et al. 2023; Galià-Camps et al. 2024; Ni et al. 2024), the limited data suggests the necessity of exploring mitochondrial genome information from more representative species. In the present study, 11 nudibranch mitogenomes were newly sequenced and analyzed. Our aims were to explore the mitogenomic structures of Nudibranchia as well as to reconstruct a more robust phylogenetic framework.

## ﻿Materials and methods

### ﻿Sample collection, species identification, and DNA extraction

The samples were collected in different locations shown in Table 1 by divers (0–40 m depths) or through trawling (40–60 m depths). Samples were first identified according to their morphological characteristics using the illustrated guides of nudibranchs (e.g., Gosliner et al. 2018; Jie 2019) as well as the references provided in WoRMS. Subsequently, the DNA samples were preserved in 95% ethanol at the Laboratory of Economic Shellfish Genetic Breeding and Cultivation Technology (**LESGBCT**) at Hainan University. Genomic DNA was extracted from the gastropods using the TIANamp Marine Animals DNA Kit (Tiangen, Beijing, China) following the manufacturer’s instructions, and the quality of extracted genomic DNA was verified on a 1% agarose gel.

**Table 1.** Collection information of newly sequenced species.

Species	Geographical locality	Depth	Collection date	Coordinates	Clean read	GenBank accession No.
*Hypselodorisbullockii*	Dadonghai, Hainan	10–20 m	1 Jun. 2023	18°22.091′N, 109°52.255′E	27,299,174	PQ035990
*Hypselodoristryoni*	Dadonghai, Hainan	5–25 m	10 Aug. 2023	18°22.091′N, 109°52.255′E	31,909,117	PQ035999
*Actinocyclus* sp.	Weitou Port, Fujian	10–40 m	4 Dec. 2023	24°51.521′N, 118°59.058′E	13,799,577	PQ035995
*Halgerdawilleyi*	Dadonghai, Hainan	10–50 m	10 Dec. 2023	18°22.091′N, 109°52.255′E	18,975,713	PQ035992
*Phyllidiellanigra*	Wuzhizhou Island, Hainan	1–6 m	17 Sep. 2023	18°31.061′N, 109°76.611′E	14,057,854	PQ035989
*Phyllidiavaricosa*	Dadonghai, Hainan	5–30 m	12 Jul. 2023	18°22.091′N, 109°52.255′E	16,617,599	PQ035998
*Arminavariolosa*	Heping Wharf, Fujian	10–60 m	16 Dec. 2023	24°44.630′N, 118°07.774′E	15,801,344	PQ035993
*Dendronotusprimorjensis*	Yellow Sea	5–20 m	4 Jan. 2024	36°02.466′N, 120°61.605′E	12,528,541	PQ035996
*Samlabicolor*	Dadonghai, Hainan	5–25 m	7 Sep. 2023	18°22.091′N, 109°52.255′E	21,846,987	PQ035991
*Sakuraeolisenosimensis*	Yellow Sea	5–30 m	14 Jan. 2024	36°02.466′N, 120°61.605′E	14,004,673	PQ035997
*Caloriamilitaris*	Luoyu Port, Fujian	5–40 m	2 Jan. 2024	25°18.104′N, 119°02.447′E	14,122,487	PQ035994

The *cox1* fragments of all samples were PCR amplified and sequenced to further determine the validity of morphological identification. The PCR primers, volumes, and conditions all followed those from Li et al. (2024). The PCR products were verified on a 1.5% agarose gel, and purified products were Sanger-sequenced by Liuhe Huada Company (Beijing, China).

### ﻿Illumina sequencing and mitochondrial genome assembly

The genomic DNA extracted from 11 nudibranch species was dispatched to Novogene (Beijing, China) for the purposes of library preparation and subsequent next-generation sequencing. A DNA library was prepared using the NEB Next Ultra DNA Library Prep Kit (NEB, USA) according to the manufacturer’s instructions, creating a library with an insert size of approximately 300 bp. Sequencing was performed on the Illumina NovaSeq 6000 platform, yielding 150 base pair paired-end reads. The number of raw reads per direction for the 11 species is provided in Table 1. Adapter sequences and low-quality reads were filtered using Trimmomatic (Bolger et al. 2014). The clean data were imported into Geneious Prime 2024.0.1 (Kearse et al. 2012) for mitochondrial genome assembly, following the strategy outlined by Irwin et al. (2021). The assembled mitochondrial genomes were submitted to GenBank with accession numbers shown in Table 1.

### ﻿Mitochondrial genome annotation and sequence analysis

Annotation of the 11 mitochondrial genomes was performed using Geneious Prime. Open reading frames (ORFs) were identified using ORF Finder (http://www.ncbi.nlm.nih.gov/orffinder), and boundaries were corrected by comparing them to genes within the same family. Boundaries of tRNAs were predicted by Mitos2 (Arab et al. 2017), and rRNA boundaries were corrected based on homologous genes in other nudibranchs. Mitochondrial genome maps were generated using CGView (Grant and Stothard 2008). Nucleotide compositions of complete mitochondrial genomes PCGs, rRNAs, and tRNA genes, as well as the genetic distance of *Hypselodorisbullockii*, *Actinocyclus* sp., and *Arminavariolosa* were calculated using MEGA X (Kumar et al. 2018). Base skewness was calculated using the formulas AT skew = (A − T) / (A + T) and GC skew = (G − C) / (G + C), where A, T, G, and C represent the frequencies of the four nucleotide bases (Perna and Kocher 1995). Codon usage among the 11 nudibranch mitochondrial genomes was analyzed using MEGA X. Comparative gene order analyses were conducted using newly sequenced mitochondrial DNA (mtDNA) data with all available mitogenomes of nudibranch mollusks from NCBI. Gene-order analysis was performed by comparing all distinct gene orders against the most common gene order (considered as the ancestral gene order of Nudibranchia; Grande et al. 2008) of Nudibranchia and visualized using Microsoft Visio 2016. To explore the selective pressures experienced by nudibranchs during evolution, we calculated the selection pressure (estimated by Ka/Ks) for the 13 PCGs using BUSTED integrated in Datamonkey Adaptive Evolution Server (Weaver et al. 2018). In addition, the phylogenetic relationships of *Hypselodorisbullockii*, *Actinocyclus* sp., and *Arminavariolosa* were constructed based on all *cox1* fragments available at NCBI (Puillandre et al. 2021).

### ﻿Phylogenetic analysis

Mitogenomes of *Hypselodorisbullockii*, *H.tryoni*, *Actinocyclus* sp., *Halgerdawilleyi*, *Phyllidiellanigra*, *Phyllidiavaricosa*, *Arminavariolosa*, *Dendronotusprimorjensis*, *Samlabicolor*, *Sakuraeolisenosimensis*, and *Caloriamilitaris* and 62 previously sequenced nudibranch mitogenomes downloaded from the GenBank database (https://www.ncbi.nlm.nih.gov/genbank) (Suppl. material 1: table S1) were used in the phylogenetic analyses to uncover the phylogenetic relationships of each nudibranch species. The outgroups were *Aplysiacalifornica* and *Aplysiadactylomela*. The dataset contained the nucleotide sequences of 13 PCGs and two rRNA genes. The PCGs were aligned separately using ClustalW integrated in MEGA X (Kumar et al. 2018), while rRNA genes were aligned separately using the default settings of MAFFT v. 7 (Katoh and Standley 2013). Ambiguously aligned positions were removed with Gblocks v. 0.91b (Castresana 2000). The different alignments were concatenated into a single dataset in Geneious Prime 2023.05. The sequences were formatted for further analysis using DAMBE5 (Xia 2013).

Nucleotide sequences of the 13 PCGs and two rRNAs were used for phylogenetic tree reconstruction using Maximum Likelihood method (ML) and Bayesian Inference method (BI). The best partition schemes were determined using PartitionFinder (Lanfear et al. 2017) under the Bayesian Information Criterion. For the 13 PCGs, the partitions tested were all genes combined; all genes separated (except *atp6*-*atp8* and *nad4*-*nad4L*); and genes grouped by subunits (*atp*, *cob*, *cox*, and *nad*). Additionally, these three partition schemes were tested considering the three codon positions separately. The two rRNA genes were analyzed with two different schemes (genes grouped or separated). The best-fit substitution models for BI analysis were also selected by PartitionFinder 2, while those for ML analysis were calculated with ModelFinder (Kalyaanamoorthy et al. 2017), as implemented in IQtree v. 1.6.12 (Nguyen et al. 2015). ML analyses were conducted using IQtree v. 1.6.12, allowing partitions to have different evolutionary rates (-spp option) and with 10,000 ultrafast bootstrap pseudo-replications (-bb option). BI analyses were performed with MrBayes v. 3.2.6 (Ronquist et al. 2012), running four simultaneous Monte Carlo Markov chains (MCMC) for 10 million generations, sampling every 1,000 generations and discarding the first 25% generations as burn-in. Two independent runs were performed to increase the chance of adequate mixing of the Markov chains and to increase the chance of detecting failure to converge, as determined by using Tracer v. 1.6. The effective sample size (ESS) of all parameters was higher than 200. The resulting phylogenetic trees were visualized in FigTree v. 1.4.2.

## ﻿Results and discussion

### ﻿Brief species descriptions (see Suppl. material 2: fig. S1 for photographs)

***Hypselodorisbullockii* (Collingwood, 1881)**

The preserved specimen measures 2.4 cm in length. The body is slender and flattened, with the foot extending posteriorly beyond the mantle edge. The mantle margin bears a narrow white line. The rhinophores are slender, with smooth cylindrical stalks and spirally lamellate clubs. The gills consist of six simple plumes, inserted into a cylindrical sheath located slightly posterior to the center of the mantle. The head is covered by the mantle and bears two small oral tentacles. The head is deep amethyst in color, gradually fading posteriorly into a paler purple with reddish tones. The rhinophores and gills gradually transition from orange at the base to a deeper shade at the tips. The foot is pale purple, deepening posteriorly, and matches the anterior mantle coloration. Commonly found in shallow reef habitats at depths of 10–20 m.

#### ﻿*Hypselodoristryoni* (Garrett, 1873)

The preserved length is 2.3 cm. The body is elliptical, broad anteriorly and tapering posteriorly, with the foot extending beyond the posterior mantle. The mantle has a purple marginal line. The rhinophores are short and thick, composed of a smooth cylindrical stalk and a lamellate club. The gills consist of multiple branched, pinnate plumes. Two small oral tentacles are present. The body is yellowish brown with large purple dorsal spots, each encircled by a white ring and a pale-yellow outer ring. A vertical white stripe runs along the rhinophores from the base to the tip. The gills are pale brown with darker brown margins. This species is often found in aggregations.

#### ﻿*Actinocyclus* sp.

The preserved specimen measures 6.1 cm. The body is oval and slightly flattened, lacking a distinct head or tail. The body is brown, with alternating paler and darker areas on the dorsum. The dorsal surface bears butterfly-shaped ridges and is covered in wart-like tubercles with purple spots. The gills are numerous and unipinnate. Commonly found in shallow reef areas, feeding on sponges lacking spicules. This specimen matches neither of the described species.

#### ﻿*Halgerdawilleyi* Eliot, 1904

The preserved length is 2.8 cm. The mantle edge is thin and broad, and the overall texture is firm and leathery. The few gill plumes exhibit various degrees of branching. Rhinophores are large, bulbous, and prominent. The body is translucent white, with many intersecting yellow ridges bearing yellow tubercles at the junctions. Alternating yellow, orange, and black lines are present between the ridges on the dorsum. The rhinophores and gills bear black marginal stripes, with black apices.

#### ﻿*Phyllidiellanigra* (van Hasselt, 1924)

The preserved length is 3.6 cm. The body is black with pink tubercles. The tubercles are typically formed by the fusion of two or three smaller tubercles and are evenly distributed on the dorsum. The rhinophores are black. The gills are located beneath the mantle margin along the lateral sides of the body and arranged in rows. This species is frequently found among coral rubble in shallow waters of the South China Sea.

#### ﻿*Phyllidiavaricosa* Lamarck, 1981

The preserved length is 3.8 cm. The body is pale blue, with opaque, orange tubercles on the dorsum separated by black longitudinal lines. The gills are located beneath the lateral mantle margin and arranged in rows. Both the tubercles and rhinophores are orange. It is commonly found on outer patch reefs and reef slopes and is one of the more frequently encountered species in the South China Sea.

#### ﻿*Arminavariolosa* (Bergh, 1904)

The preserved length is 6.7 cm. The dorsum is pale orange, with numerous, spaced, longitudinal white lines running along the ridges, ornamented with pearl-like, white tubercles. A white median line extends anteriorly and fades at midbody. The rhinophores are white and relatively short. The foot is broad, with wide, angular anterior expansions. The oral veil bears a scalloped margin. The gills are red, arranged in rows beneath the lateral mantle margin.

#### ﻿*Dendronotusprimorjensis* Martynov, Sanamyan & Korshunova, 2015

The preserved length is 1.8 cm. The body is translucent, laterally compressed, with a narrow foot and short tail. The dorsum is covered with branched gills. The body and gills are translucent, with opaque, pale-brown spots. The gill apices are brown, and internal digestive gland branches are visible through the body wall.

#### ﻿*Samlabicolor* (Kelaart, 1858)

The preserved length is 2.8 cm. The body is slender, with a small head. The oral tentacles are twice the length of the rhinophores and are curved and conical. The cerata are of moderate size, narrow, and pointed, arranged into seven paired clusters, each containing two or three cerata. Ceratal size decreases posteriorly, with the smallest near the tail, and are pale blue, with orange rings near the tips and pale purple apices.

#### ﻿*Sakuraeolisenosimensis* (Baba, 1930)

The preserved length is 2.1 cm. The oral tentacles and rhinophore bases are white. The rest of the body is translucent white, with internal organs faintly visible through the body wall, appearing dark orange-yellow. The cerata are slender, with visible brown digestive gland branches. The head, rhinophores, and dorsal cerata are orange-yellow, with scattered white dots across the dorsal surface and cerata.

#### ﻿*Caloriamilitaris* (Alder & Hancock, 1864)

The preserved length is 3.1 cm. The body is translucent white with a bright-orange median line forking and extending onto the oral tentacles. The body tapers posteriorly, terminating in a narrow and pointed tail. The oral tentacles are 1.5 times the length of the rhinophores. The rhinophores and oral and propodial tentacles transition from their bases to tips with orange-red lines and pale-yellow apices. The cerata are black and orange and tipped with yellow, with color transitioning from the base to the apex with a unilateral orange-red marginal line. A pair of short red longitudinal lines extend along each body side, fading near the midbody.

### ﻿Characteristics of nudibranch mitochondrial genomes

Nudibranchs exhibit rich species diversity, but the number of mitochondrial genomes available on GenBank is limited, hindering phylogenetic studies of the species. In this study, mitochondrial genomes from nine families were sequenced and analyzed. This study found that the mitochondrial genome size of all nudibranch species is slightly smaller compared to that of some gastropod groups, such as Nassariidae and Strombidae (Yang et al. 2021; Li et al. 2022b), which could be attributed to fewer non-coding regions, gene overlaps, or a reduction in gene sizes across all species (Sevigny et al. 2015). The newly annotated nudibranch order exhibits an overall negative AT skew and a positive GC skew, with gene arrangements similar to those recorded in the mitochondrial genomes of other nudibranch animals. Typically, gene arrangement in nudibranchs is conservative with little variation (Do et al. 2022; Galià-Camps et al. 2024).

**Figure 1.**
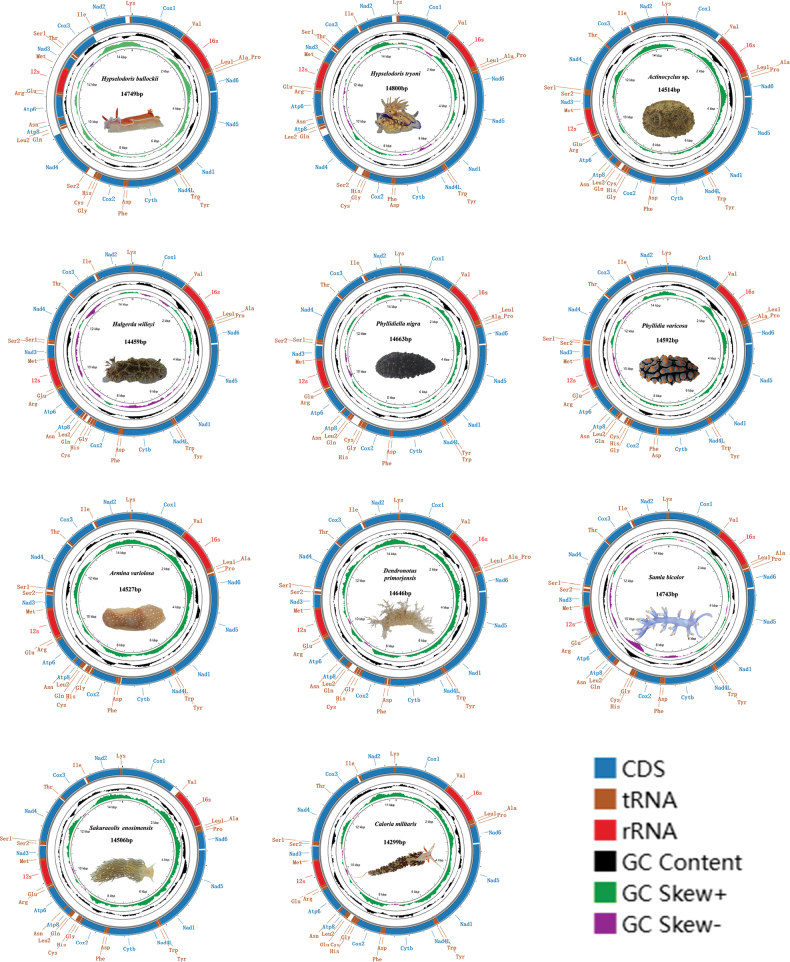
Mitochondrial genomic maps of 11 nudibranch species sequenced in this study.

**Figure 2.**
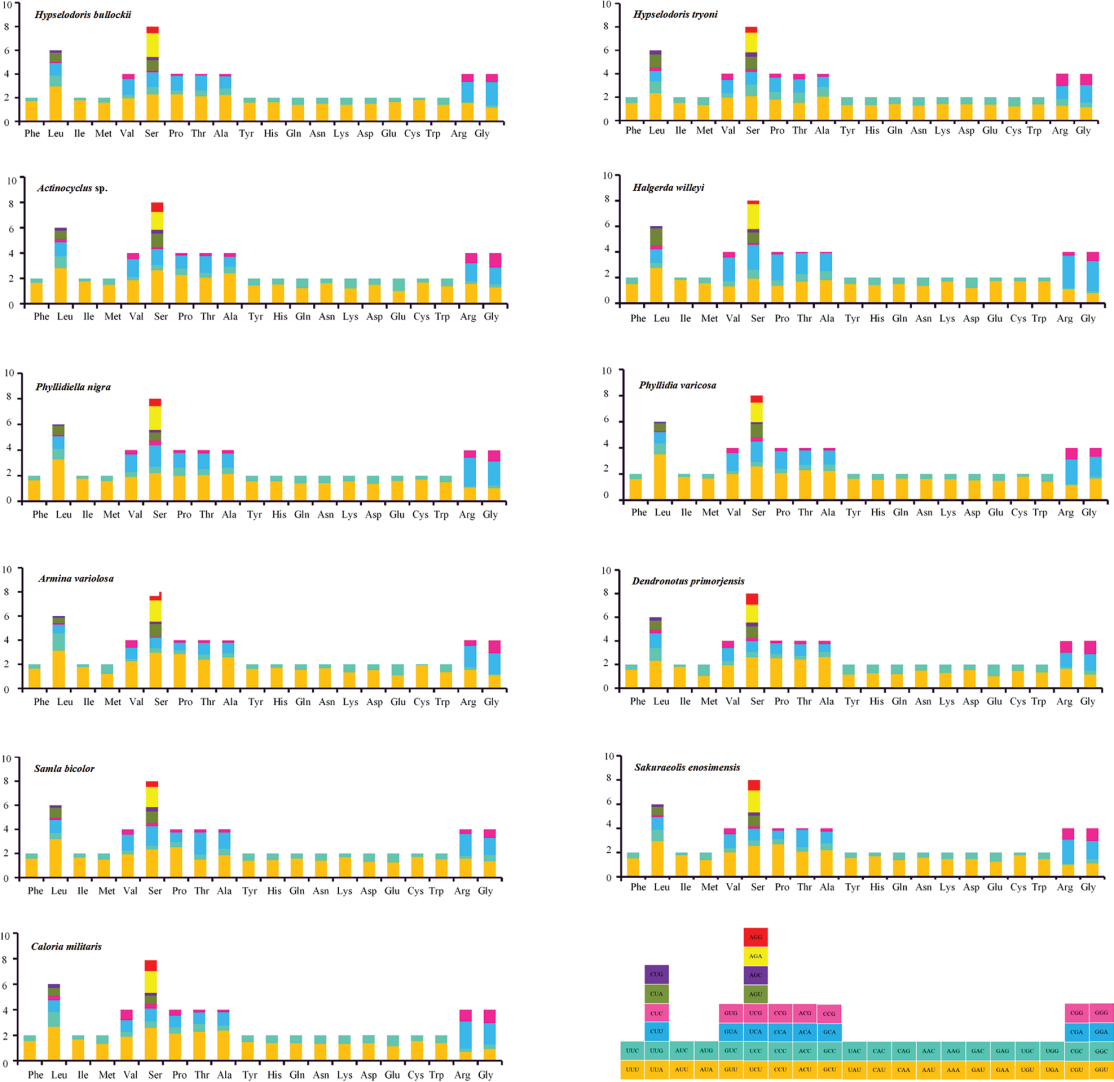
Relative synonymous codon usage (RSCU) for 11 nudibranch species.

**Figure 3.**
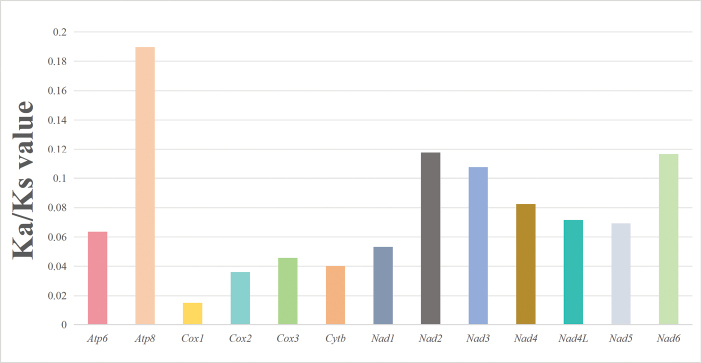
The Ka/Ks ratios of the 13 different mitochondrial genes in nudibranchs.

The mitochondrial genomes of 11 nudibranch species were newly sequenced and characterized (Fig. 1). Genome size ranges from 14,299 bp to 14,880 bp (Suppl. material 1: table S2). Except for *Samlabicolor* and *Arminavariolosa*, which have an additional *tRNA-Ser*; all other nudibranch mitochondrial genomes comprise 13 PCGs, 2 rRNA genes, 22 tRNA genes, and a presumed origin of replication, which is located between *tRNA-Phe* and *cox3* (Cunha et al. 2009; Arquez et al. 2014). Of these 37 genes, 24 are encoded on the heavy strand and 13 on the light strand. Moreover, the overall A+T content of these mitochondrial genomes is high (Suppl. material 1: table S3), ranging from 64.2% (*Dendronotusprimorjensis*) to 70.6% (*Phyllidiavaricosa*), still in accordance with the characteristics of molluscan mitochondrial genomes (Grande et al. 2008; Xu et al. 2012; Arquez et al. 2014; Sun et al. 2015; Galià-Camps et al. 2024, 2025). The values for AT skew and GC skew are from −0.189 (*Arminavariolosa*) to −0.025 (*Halgerdawilleyi*) and from −0.034 (*Halgerdawilleyi*) to 0.237 (*Arminavariolosa*). Codon usage preferences, influenced by mutations, selective pressure, and genetic drift, are crucial for understanding genomic evolution. The 11 nudibranch species encode 3,610–3,647 amino acids (Fig. 2). Among these mitogenomes, leucine is the most frequently encoded amino acid, followed by serine, while glutamine, arginine, and cysteine are the least encoded ones, which is consistent with other nudibranch species (Do et al. 2022). The most and least frequently used codons are UUA and CGC, respectively. In the present 11 mitochondrial genomes, the codon usage shows a bias toward amino acids encoded by A+T-rich codons, such as UUA-*Leu*, AUU-*Ile*, UUU-*Phe*, and AUA-*Met.* Among the 22 amino acids, nine (*Leu1*, *Val*, *Ser2*, *Pro*, *Thr*, *Ala*, *Arg*, *Ser1*, and *Gly*) have four codons each, while the remainder have two codons each. In the 11 nudibranch mitochondrial genomes analyzed herein, the RSCU values for most amino acids are very similar, although some individual variations may exist among different species, hinting at similar functions of the gene among different families of Nudibranchia (Fig. 2). Furthermore, these species preferentially have codons ending in A or T, aligning with their preference for AT-rich codons. The primary factors influencing codon preference include mutational pressure, genetic drift, and natural selection (Parvathy et al. 2022). Mutational pressure within animals is the main evolutionary force driving the increase in A+T or G+C content (Jakovlic et al. 2021). The Ka/Ks value (the ratio of non-synonymous to synonymous substitutions) is widely used to assess the selective pressures and evolutionary relationships (Zhu et al. 2017). The Ka/Ks ratio ranged from 0.0151 (*cox1*) to 0.1897 (*atp8*), suggesting that they are evolving under purifying selection (Fig. 3). Among them, the *atp8* and *nad2* genes have relatively higher Ka/Ks values, while the *cox1* and *cox2* genes have lower ones.

Protein-coding genes (PCGs). In protein-coding genes (PCGs), the trend towards negative AT skew and positive GC skew becomes more pronounced. The start and stop codons and the lengths of the PCGs are listed in Table 2. Most PCGs start with ATN, although some begin with TTG or GTG, similar to most mollusk mitogenomes (Ma et al. 2023). All PCGs end with TAA or TAG, except for the *cox2* of *Caloriamilitaris* and *nad3* of *Dendronotusprimorjensis* that display an incomplete stop codon T. The truncated stop codons (T and TA) have been reported in metazoan mtDNA (Yu and Li 2012), and it is likely modified post-transcriptionally to a complete TAA through polyadenylation.

**Table 2.** Start and stop codons of nudibranch mitochondrial PCGs of the newly sequenced species.

	*Hypselodorisbullockii*	*Hypselodoristryoni*	*Actinocyclus* sp.	*Halgerdwilleyi*	*Phyllidiellanigra*	*Phyllidiavaricosa*	*Arminavariolosa*
*atp*6	675(**ATA**/TAG)	666(ATG/TAA)	663(ATG/TAA)	678(ATG/TAA)	669(**GTG**/TAA)	675(ATG/TTA)	663(ATG/TAA)
*atp8*	156(ATG/TAA)	156(ATG/TAG)	156(ATG/TAA)	156(ATG/TAA)	156(ATG/TAA)	156(ATG/TAA)	186(ATG/TAA)
*cox1*	1533(ATG/TAA)	1533(ATG/TAA)	1530(ATG/TAG)	1533(ATG/TAA)	1530(ATG/TAA)	1530(ATG/TAA)	1530(**GTG**/TAA)
*cox2*	675(ATG/TAA)	675(ATG/TAG)	675(ATG/TAA)	675(ATG/TAG)	678(ATG/TAA)	678(ATG/TAA)	675(ATG/TAA)
*cox3*	780(ATG/TAG)	780(ATG/TAG)	780(ATG/TAG)	780(ATG/TAG)	780(ATG/TAG)	780(ATG/TAG)	780(ATG/TAA)
*cytb*	1128(ATG/TAG)	1128(ATG/TAG)	1128(ATG/TAA)	1128(ATG/TAA)	1131(ATG/TAG)	1128(ATG/TAG)	1134(ATG/TAA)
*nad1*	897(**GTG**/TAA)	912(**GTG**/TAG)	915(ATG/TAA)	912(**GTG**/TAA)	915(ATG/TAA)	915(ATG/TAA)	912(ATG/TAG)
*nad2*	942(ATG/TAG)	939(ATG/TAA)	936(ATG/TAA)	933(ATG/TAG)	969(ATG/TAA)	954(ATG/TAG)	942(ATG/TAG)
*nad3*	354(ATG/TAA)	354(ATG/TAA)	354(ATG/TAA)	354(ATG/TAA)	390(**GTG**/TAA)	354(ATG/TAA)	354(ATG/TAA)
*nad4*	1314(ATG/TAA)	1314(**TTG**/TAG)	1344(**GTG**/TAG)	1326(ATG/TAG)	1317(ATG/TAA)	1347(ATG/TAG)	1314(ATG/TAA)
*nad4l*	294(**TTG**/TAA)	288(ATG/TAA)	294(**GTG**/TAA)	294(**GTG**/TAA)	294(**GTG**/TAA)	294(**GTG**/TAA)	294(**TTG**/TAA)
*nad5*	1623(ATG/TAG)	1680(ATG/TAG)	1623(ATA/TAG)	1623(ATA/TAG)	1623(ATG/TAG)	1623(ATA/TAG)	1689(ATG/TAA)
*nad6*	459(**TTG**/TAA)	468(**TTG**/TAA)	468(**TTG**/TAA)	468(**TTG**/TAA)	471(**TTG**/TAA)	474(**TTG**/TAA)	468(**TTG**/TAA)
	***Dendronotusprimorjensis***	***Samlabicolor***	***Sakuraeolisenosimensis***	***Caloriamilitaris***			
*atp6*	666(ATG/TAG)	663(ATG/TAA)	690(ATG/TTA)	663(ATG/TAA)			
*atp8*	180(ATG/TAA)	153(ATG/TAA)	186(ATG/TAA)	186(ATG/TAA)			
*cox1*	1530(ATG/TAG)	1533(ATG/TAA)	1548(**GTG**/TAA)	1530(**GTG**/TAA)			
*cox2*	675(ATG/TAA)	675(ATG/TAA)	693(ATG/TAA)	676(ATG/T--)			
*cox3*	780(ATG/TAA)	780(ATG/TAA)	780(ATG/TAA)	780(ATG/TAA)			
*cytb*	1206(ATG/TAA)	1122(ATG/TAA)	1125(ATG/TAA)	1122(ATG/TAG)			
*nad1*	918(**TTG**/TAG)	915(**GTG**/TAA)	912(ATG/TAA)	918(**TTG**/TAG)			
*nad2*	936(ATG/TAG)	936(ATG/TAG)	945(**GTG**/TAG)	939(ATG/TAA)			
*nad3*	408(ATG/T--)	375(**GTG**/TAA)	375(**TTG**/TAA)	354(**TTG**/TAG)			
*nad4*	1314(ATG/TAA)	1314(ATG/TAA)	1314(ATG/TAA)	1386(**GTG**/TAG)			
*nad4l*	294(**TTG**/TAA)	288(ATG/TAA)	294(**GTG**/TAA)	294(**GTG**/TAA)			
*nad5*	1623(ATG/TAG)	1680(ATG/TAG)	1623(ATA/TAG)	1623(ATA/TAG)			
*nad6*	459(**TTG**/TAA)	468(**TTG**/TAA)	468(**TTG**/TAA)	468(**TTG**/TAA)			

**Figure 4.**
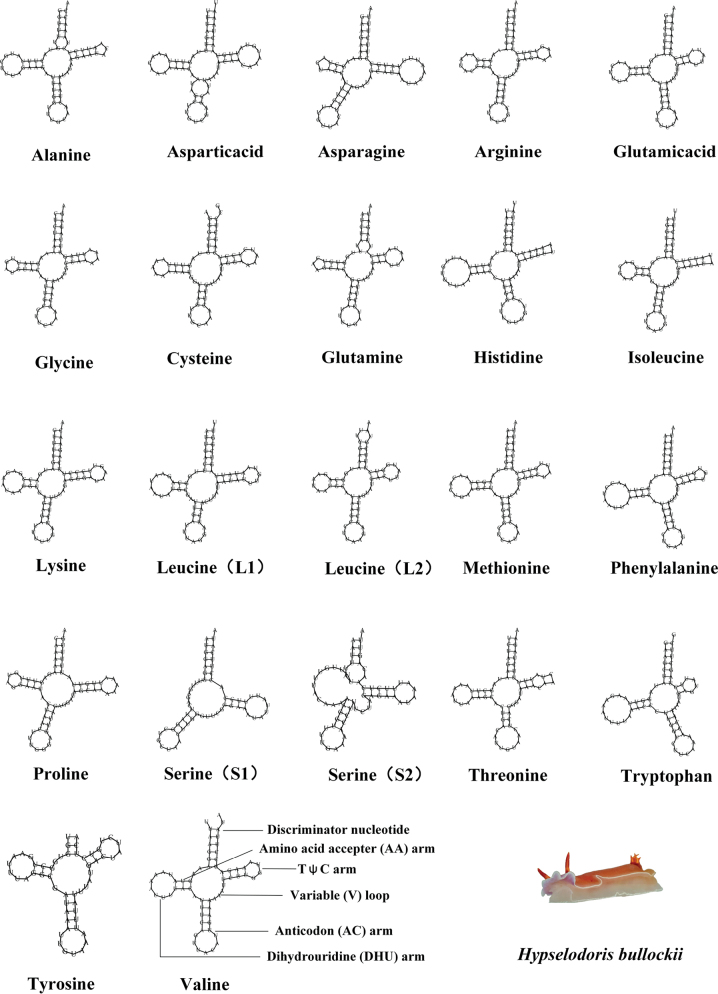
Inferred secondary structures of 22 transfer RNAs (tRNAs) represented by *Hypselodorisbullockii*.

Transfer RNA (tRNA) genes. Most metazoan mitochondrial genomes contain 22 tRNAs, with two copies each of *tRNA-Leu* and *tRNA-Ser*, and one copy of the other 18 tRNAs (Podsiadlowski et al. 2008). In this study, the newly sequenced mitochondrial genomes of 11 nudibranch species were all found to contain 22 tRNA genes (Fig. 4). Upon predicting the secondary structures of these tRNAs, it was observed that 21 of them could fold into the classic cloverleaf secondary structure except a specific type of tRNA, *tRNA-Ser* (AGN), that lacked the dihydrouridine (DHU) arm (Fig. 4). This phenomenon is not unique and has been previously reported in other metazoans (Wolstenholme 1992).

Ribosomal RNA (rRNA) genes. The accurate boundaries of ribosomal RNAs were challenging to determine before the use of mitochondrial transcript mapping (He et al. 2011). The large (16S rRNA) and small (12S rRNA) rRNA genes are encoded by the H and L strands, respectively (Suppl. material 1: table S2). The 12S rRNA gene, located between *tRNA-Glu* and *tRNA-Val*, varies in length from 700 bp (*Sakuraeolisenosimensis*) to 743 bp (*Phyllidiellanigra*). The 16S rRNA gene, situated between *tRNA-Val* and *tRNA-Leu1* (CUA), ranges from 949 bp (*Sakuraeolisenosimensis*) to 1,143 bp (*Phyllidiavaricosa*). The overall AT and GC skew of rRNAs and tRNAs are irregular, with positive and negative values detected in the corresponding genes (Suppl. material 1: table S3).

**Figure 5.**
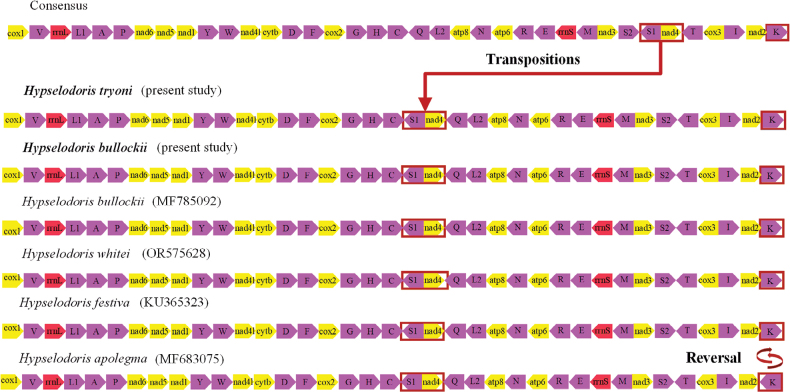
Mitochondrial gene rearrangements of nudibranchs.

Gene rearrangement. Gene rearrangement has been observed in *H.bullockii*, *H.apolegma*, *H.festiva*, *H.tryoni*, *H.whitei* and within Chromodorididae (*H.bullockii* and *H.apolegma*, Lin et al. 2019; *H.festiva*, Karagozlu et al. 2016), where *tRNA-Ser* and *nad4* are rearranged between *tRNA-Cys* and *tRNA-Gln* (Lin et al. 2019) (Fig. 5). A recent study identified a cytb gene rearrangement in *D.temarana* and *D.limbata* (Galià-Camps et al. 2024). However, current data only confirm gene rearrangements in *Hypselodoris* and *Dendrodoris*. Given the limited number of studied species within Nudibranchia, it remains unclear whether such rearrangements are specific to *Hypselodoris* and *Dendrodoris* or occur more broadly across other genera within Nudibranchia. This warrants further investigation to clarify the distribution and evolutionary significance of these genomic rearrangements.

### ﻿Phylogenetic analysis

**Figure 6.**
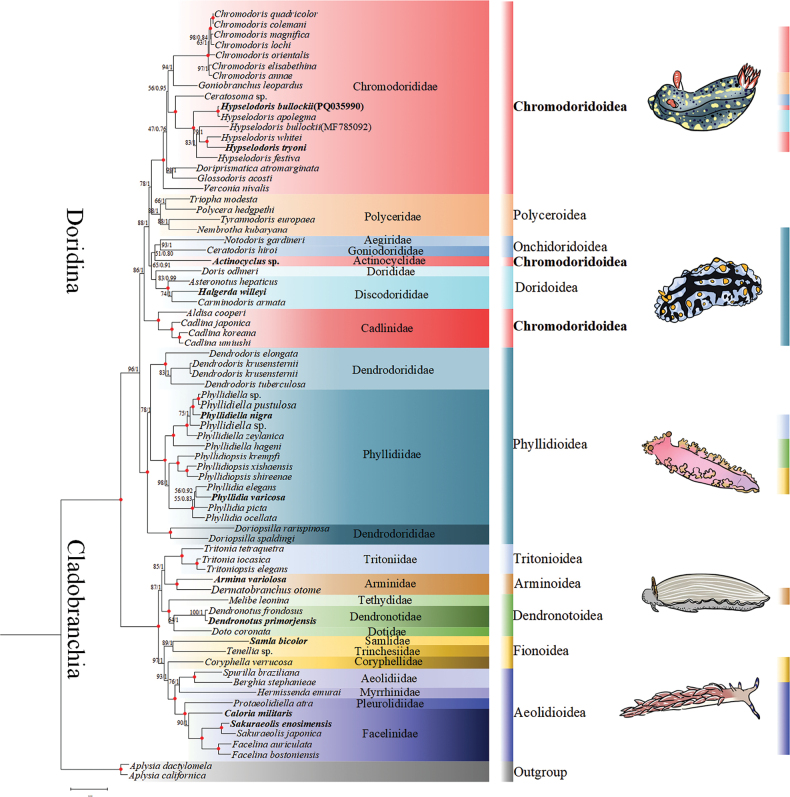
Phylogenetic relationships based on concatenated 13 mitochondrial PCGs and two mitochondrial rRNA genes, with *Aplysiacalifornica* and *A.dactylomela* as outgroups. The first number on each node is the bootstrap proportion (BP) of the maximum likelihood (ML) analysis. The second number is the Bayesian posterior probability (PP). Nodes with maximum statistical support (BP = 100; PP = 1) are marked with red solid circles. The 11 newly sequenced mitochondrial genomes and the paraphyletic group Chromodoridoidea are in bold.

Phylogenetic relationships of Nudibranchia were reconstructed based on the nucleotide sequences of the 13 PCGs plus two rRNA genes using ML and BI methods (Fig. 6). The dataset was 11,909 bp in length. According to the BIC, the best partition scheme for the PCGs was the one combining genes by subunits but analyzing each codon position separately. For the rRNA genes, the best partition scheme was the one combining rrnS and rrnL genes together. The best fit models for different partitions are shown in Suppl. material 1: table S4. Both ML (-lnL = 378,113.212) and BI (-lnL = 37,570.170 for run 1; -lnL = −375,705.210 for run 2) analyses arrived at almost identical topologies. Nudibranchs consist of two highly supported branches (posterior probability value of BI = 1; bootstrap proportion value of ML = 100) (Fig. 6), which is consistent with the current classification that divides Nudibranchia into Doridina and Cladobranchia (MolluscaBase Eds 2025a). The suborder Doridina currently includes 1,524 species, exhibiting a rich diversity (MolluscaBase Eds 2025b). Phylogenetic analyses consistently support the suborder Doridina as a monophyletic group (Wollscheid and Wägele 1999; Wollscheid-Lengeling et al. 2001; Gobbeler and Klussmann-Kolb 2010). It is generally recognized that dorids can be divided into two types: cryptobranchs (with a gill cavity) and phanerobranchs (without a gill cavity) (Korshunova et al. 2020; Do et al. 2022), although the monophyly of these groups remains controversial (Mahguib and Valdés 2015). Based on the obtained phylogenetic relationships, it was found that within the group Doridina, the superfamilies Polyceroidea, Onchidoridoidea, Doridoidea, and Phyllidioidea are demonstrated to be monophyletic, whereas Chromodoridoidea is considered paraphyletic if *Actinocyclus* sp. and Cadlinidae are included (Fig. 6), which is consistent with the results of a previous study (Korshunova et al. 2020). The non-monophyly of Chromodoridoidea has also been revealed in previous studies based on concatenated fragments and complete mitochondrial genomes (e.g., Hallas et al. 2017; Breslau 2021; Do et al. 2022), due to the distant positions between Chromodorididae. Historically, *Cadlina* of Cadlinidae was thought to be closely related to members of Chromodorididae such as *Chromodoris* and *Hypselodoris* in terms of radula and reproductive structures (Johnson 2011). However, chromodorids lack penial spines, spicules in the mantle tissues, and tubercles on the mantle surface compared with *Cadlina* (Johnson 2011; Korshunova et al. 2020). At the same time, due to significant differences in the details of the central and lateral teeth, as well as disparities in the reproductive system and gill apparatus, *Cadlinasagamiensis* has been separated from the genus *Cadlinella* and placed into a newly established genus, *Showajidaia*, which better accommodates the morphological differences observed (Korshunova et al. 2020). Geographically, Chromodorididae is predominantly found in tropical and subtropical waters, whereas Cadlinidae is common in temperate and polar waters (Korshunova et al. 2020). The molecular, morphological, and ecological differences between the two families support that Cadlinidae should be removed from Chromodoridoidea. Even though *Actinocyclus* sp. possesses typical morphological features of Chromodoridoidea, it has not clustered with either Chromodorididae or Cadlinidae in the current mitogenomic phylogeny nor in previous studies based on concatenated molecular data (*COI* + *16S* + *28S* + *18S*) or RNA-Seq data (Korshunova et al. 2020; Breslau 2021). According to a previous study, *Actinocyclus* sp. is a sister group to a clade uniting Dorididae and Discodorididae, characterized by a radula with many lateral teeth and a clearly visible buccal commissure (Lima 2016). However, our study showed Actinocyclidae clustered with Aegiridae + Goniodorididae (BP = 51) and then grouped with Dorididae + Discodorididae (BP = 65; PP = 0.80), although with low support values.

Within Onchidoridoidea, the classification has been revised based on a series of molecular phylogenetic studies (Hallas and Gosliner 2015), leading to the establishment of the new genus *Idaliadoris* (Furfaro et al. 2022), as well as the description of two new species (Moles et al. 2017; Furfaro et al. 2022). The monophyly of the family Goniodorididae has also been supported (Paz-Sedano et al. 2022). However, the internal relationship within Goniodorididae has received less attention, and the monophyly of genera remains unexamined (Pola et al. 2019; Paz-Sedano et al. 2021, 2022). Meanwhile, molecular and morphological data support the grouping of Discodorididae + Dorididae and Aegiridae + Goniodorididae (Do et al. 2022) are consistent with the present study.

The Phyllidioidea formed a monophyletic clade (BP = 100; PP = 1) (Fig. 6), corroborating previous studies based on substantial morphological and anatomical evidence (Valdés and Gosliner 1999; Valdés 2003). Compared with other dorids, Phyllidioidea possesses several morphological synapomorphies such as the lack of a radula and modified foregut adapted for suctorial feeding, both of which are complex adaptations (Valdés 2003). Phylogenetic analyses show that the *Dendrodoris* clade of Dendrodorididae has a closer sister-group relationship with Phyllidiidae, and together they form a clade with *Doriopsilla*, suggesting that Dendrodorididae is not a monophyletic group. Species within this family generally lack distinct morphological features—most notably the radula, which is one of the key taxonomic characters for distinguishing and defining nudibranch species, posing significant challenges for taxonomy identified accelerated mutation rates in the mitochondrial genomes of Dendrodorididae, potentially linked to respiratory adaptation, offering a novel explanation for the taxonomic difficulties (Galià-Camps et al. 2024). The phylogenetic relationships within this family remain unresolved. Future studies should expand taxon sampling and integrate morphological (e.g., radula) and molecular data (nuclear, mitochondrial, and transcriptomic) for more robust phylogenetic reconstruction.

The monophyletic suborder Cladobranchia includes Tritonioidea, Arminoidea, Dendronotoidea, and Aeolidioidea (Fig. 6). Within Cladobranchia, Dendronotoidea is sister to the Tritonioidea + Arminoidea (BP = 87; PP = 1). However, consistent with the findings of Karmeinski et al. (2021), Arminoidea and Tritonioidea are each represented by only a single genus in this study. As a result, the monophyly of these groups requires further validation through more comprehensive genomic analyses in the future. The close relationship between Tritonioidea and Arminoidea has also been supported by molecular data (Wollscheid-Lengeling et al. 2001) as well as dietary habits (Goodheart et al. 2017).

Within the Aeolidioidea, Samlidae and Trinchesiidae form a sister group relationship (BP = 89; PP = 1). However, Fionoidea is demonstrated to be polyphyletic, as Coryphellidae within it clusters with Aeolidioidea (BP = 93; PP = 1), with only the latter being supported as monophyletic (Wollscheid and Wägele 1999). This result is consistent with the findings of Karmeinski at al. (2021), whose phylogenetic tree also indicated the polyphyly of Fionoidea, primarily due to the positions of *Flabellinaaffinis* and *Embletoniapulchra*. The phylogenetic tree indicates that the polyphyly of Fionoidea is attributed to the position of Coryphellidae. In recent studies, four new genera within Coryphellidae have been identified, and phylogenetic analyses based on combined gene fragments have been conducted (Ekimova et al. 2022). However, the phylogenetic analysis in this study includes only one mitochondrial genome from Coryphellidae. Therefore, further revision of the systematics of this family is necessary, incorporating additional mitochondrial genomes to refine its taxonomic relationships.

### ﻿Naming confusion of the species *Hypselodorisbullockii*, Actinocycluscf.verrucosus, and *Arminavariolosa*

To investigate potential issues of confusion in species identification within Nudibranchia, originally two species were selected for dataset construction: *Dendrodoriskrusensternii* and *Hypselodorisbullockii*. The phylogenetic tree revealed that the sequences of two *Dendrodoriskrusensternii* were clustered into one branch, while the two *H.bullockii* (PQ035990; MF785092) sequences were not clustered together (Fig. 6). To further validate this observation, the *cox1* sequence of the newly sequenced *H.bullockii* (collected from Hainan, China) was aligned and analyzed together with the nine available *H.bullockii* sequences from NCBI (PQ035990–Dadonghai, Hainan; MF785092–Indonesia; EU982743–Philippines; JQ727888–Japan; MG645568–Marshall islands; MG645569–Philippines; MG645570–Philippines; MG645571–Philippines; MG645572–Philippines (Suppl. material 1: table S1). The sequence MF785092 from Indonesia was separated from all other *H.bullockii* sequences (Suppl. material 2: fig. S2) and an average genetic distance of 0.1291 was detected between MF785092 and other *H.bullockii* sequences through *COI*-based genetic distance analysis, being significantly greater than the intraspecific distance range (0–0.0081; Suppl. material 1: table S5). Significant genetic distance differences exist among the samples, and therefore further discussion is needed regarding the species identification. *Hypselodorisbullockii* was described as having a head with a deep amethyst hue, fading from behind the dorsal tentacles to a paler amethyst and a reddish tint on the back. The foot was described as pale amethyst, darkening towards the posterior, where it matched the color intensity of the anterior mantle, with a distinct narrow white margin (Collingwood 1881; type locality: South China Sea). Additionally, *H.bullockii* was described and illustrated from the Indian Ocean region (Yonow 2012), and the newly sequenced *H.bullockii* (collected from Hainan, China) was found to match with the original morphological description and is also consistent with the Indian Ocean record.

This discrepancy led us to further examine the species identification of the newly sequenced *Actinocyclus* (PQ035995), and a comparison and phylogenetic analysis were conducted with five *Actinocyclus cox1* sequences from NCBI (OQ573576–China; MF958438–Kauai, Hawaii, USA; EF535108–Mooloolaba, Queensland, Australia; MW277700–Kaneohe Bay, Hawaii, USA; MW278846–Kaneohe Bay, Hawaii, USA; see Suppl. material 1: table S1). The results showed that *Actinocyclus* (PQ035995) clustered with an unpublished Actinocycluscf.verrucosus (OQ573576; Suppl. material 2: fig. S3), with a genetic distance of 0.0013 (Suppl. material 1: table S6), supporting it as the same species with both being different from *A.verrucosus*. Our specimen lacks the typical dense black dorsal eye spots of *A.verrucosus* and its morphological characteristics do not match; therefore, it is identified as *Actinocyclus* sp.

During the identification of *Arminavariolosa*, a sequence misidentified as *A.variolosa* (MW940328) was detected in the NCBI database. To assess this issue, sixteen *cox1* sequences of *Armina* species were retrieved from the database, and a phylogenetic tree was subsequently constructed: the results showed that the newly sequenced *A.variolosa* (PQ035993) did not cluster with the previously sequenced *A.variolosa* (MW940328) (Suppl. material 2: fig. S4), with a genetic distance of 0.231 (Suppl. material 1: table S7). Our specimen is large-bodied and matches the description by Bergh (1904) and Liu (2008): the head veil is semicircular, the rhinophores are perfoliate, the mantle is narrow, and the mid-dorsum is covered with thick granules arranged longitudinally. The head veil is orange-yellow, and the rhinophores are pale orange-yellow. This specimen was collected from Hainan, China, while the type locality noted in the original was “China Sea”, which further supports the accuracy of its taxonomic identification. Jensen (1997) also described *Arminavariolosa* (from Hong Kong) as being distinctly larger than other nudibranchs and sharing the same external morphological features, including small white warts scattered across the dorsum and red gill blades. Based on morphological characteristics and locality data, we conclude that the newly sequenced specimen belongs to *Arminavariolosa*, and that the other sequence is misidentified.

Therefore, in species identification and classification, it is essential to integrate morphological characteristics, molecular data, and biogeographical locations to ensure accuracy and consistency.

## ﻿Conclusions

The 11 newly sequenced mitochondrial genomes provide a novel perspective for investigating the phylogenetic relationships within Nudibranchia. The main findings are as follows:

1. by integrating all available Nudibranchia data from NCBI, it has been confirmed that genomic rearrangements are specifically observed in *Hypselodoris* (this study) and *Dendrodoris* (Galià-Camps et al. 2024);
2. the non-monophyly of Chromodoridoidea suggested by Do et al. (2022) is further supported by phylogenetic analyses based on mitochondrial genomes;
3. Coryphellidae is clustered within Aeolidioidea, reaffirming the monophyly of Fionoidea as suggested by Karmeinski at al. (2021);
4. misidentification of nudibranch species has been observed in public databases (e.g., *Hypselodorisbullockii*, *Arminavariolosa*);
5. this study highlights the need for incorporating additional mitogenomic data alongside morphological evidence to further revise the systematics of Nudibranchia.

## ﻿Additional information

### Use of AI

No use of AI was reported.

### Author ORCIDs

Yi Yang https://orcid.org/0000-0002-8911-8501

### Data availability

Data presented in this study are openly available in the GenBank repository with accession numbers: PQ035989–PQ035999.

## Supplementary materials

Supplementary material 1 Supplementary tables S1–S7 Data type pdf Explanation note **table S1.** Taxonomic information, GenBank accession numbers (NCBI), and European Nucleotide Archive (ENA) (PRJEB75450) experiment accession numbers of the sequences used in this study are provided. Sequences obtained in this study are marked in bold. **table S2.** Structural features of Nudibranchia mitochondrial genomes. **table S3.** AT and GC Skew rate of Nudibranchia mitochondrial genomes. **table S4.** Best fit partitions and substitution models. **table S5.** Genetic distance of *Hypselodorisbullockii cox1* in NCBI. **table S6.** Genetic distance of *Actinocyclus cox1* in NCBI **table S7.** Genetic distances of *Armina cox1* in NCBI https://binary.pensoft.net/file/1367659This dataset is made available under the Open Database License (http://opendatacommons.org/licenses/odbl/1.0/). The Open Database License (ODbL) is a license agreement intended to allow users to freely share, modify, and use this Dataset while maintaining this same freedom for others, provided that the original source and author(s) are credited.Wanying Li, Fengping Li, Mingjie Liu, Aimin Wang, Chunsheng Liu, Yi Yang

Supplementary material 2 Supplementary figures S1–S4 Data type pdf https://binary.pensoft.net/file/1367660This dataset is made available under the Open Database License (http://opendatacommons.org/licenses/odbl/1.0/). The Open Database License (ODbL) is a license agreement intended to allow users to freely share, modify, and use this Dataset while maintaining this same freedom for others, provided that the original source and author(s) are credited.Wanying Li, Fengping Li, Mingjie Liu, Aimin Wang, Chunsheng Liu, Yi Yang
