# Supplementary material for: ﻿Characterization of 11 complete mitochondrial genomes in Nudibranchia (Mollusca, Gastropoda)
Source: Zookeys. 2025 Jul 4;1244:61–86. doi: 10.3897/zookeys.1244.139617 (PMC12254827; doi:10.3897/zookeys.1244.139617)
Supplement: Supplementary material 1 — Supplementary tables S1–S7 [file zookeys-1244-061_article-139617__-s001.pdf]

Table S1. Taxonomic information, GenBank accession numbers (NCBI), and European Nucleotide Archive (ENA) (PRJEB75450) experiment accession numbers of the sequences used in this study are provided. Sequences obtained in this study are marked in bold.

Suborder	Superfamily	Family	Genus	Species	mtDNA	Cox1	Source
Doridina	Phyllidioidea	Phyllidiidae	*Phyllidiopsis*	*Phyllidiopsis shireenae*	OP464905		Unpublished
				Phyllidiopsis xishaensis	OP464904		Unpublished
				Phyllidiopsis krempfi	MT726194		Kim et al. (2021)
			Phyllidiella	*Phyllidiella zeylanica*	OP464903		Unpublished
				Phyllidiella sp1.	OP464900		Unpublished
				Phyllidiella pustulosa	OP464899		Liu et al. (2024)
				Phyllidiella hageni	OP464898		Unpublished
				Phyllidiella sp2.	MG822789		Liu et al. (2023)
				Phyllidiella nigra	**PQ035989**		This study
			Phyllidia	*Phyllidia picta*	OP464895		Unpublished
				Phyllidia ocellata	OP464894		Xiang et al. (2016a)
				Phyllidia elegans	OM273027		Li et al. (2022)
				Phyllidia varicosa	**PQ035998**		This study
		Dendrodorididae	*Dendrodoris*	*Dendrodoris krusensternii*	OR463910		Lee et al. (2024)
				Dendrodoris krusensternii	ERX12443352		Galià-Camp et al. (2024b)
				Dendrodoris tuberculosa	ERX12443351		Galià-Camp et al. (2024b)
				Dendrodoris elongata	ERX12443353		Galià-Camp et al. (2024b)
			Doriopsilla	*Doriopsilla rarispinosa*	ERX12443354		Galià-Camp et al. (2024b)
				Doriopsilla spaldingi	ERX12443355		Galià-Camp et al. (2024b)
	Chromodoridoidea	Chromodorididae	*Chromodoris*	*Chromodoris annae*	OR525537		Lin et al. (2017)

		Chromodoris colemani	OR497842		Unpublished
		Chromodoris magnifica	DQ991931		Medina et al.(2011)
		Chromodoris quadricolor	KU317089		Xiang et al.(2016b)
		Chromodoris orientalis	MH550543		Yu et al.(2018)
		Chromodoris elisabethina	OR463901		Unpublished
		Chromodoris lochi	OR161407		Unpublished
	Hypselodoris	*Hypselodoris festiva*	KU365323		Karagozlu et al.(2016a)
		Hypselodoris apolegma	MF683075		Lin et al.(2019)
		Hypselodoris whitei	OR575628		Unpublished
		Hypselodoris bullockii	**PQ035990**		This study
		Hypselodoris bullockii	MF785092		Lin et al.(2019)
		Hypselodoris bullockii		EU982743	Johnson RF (2012)
		Hypselodoris bullockii		JQ727888	Johnson RF(2012)
		Hypselodoris bullockii		MG645568	Epstein et al.(2019)
		Hypselodoris bullockii		MG645569	Epstein et al.(2019)
		Hypselodoris bullockii		MG645570	Epstein et al.(2019)
		Hypselodoris bullockii		MG645571	Epstein et al.(2019)
		Hypselodoris bullockii		MG645572	Epstein et al.(2019)
		Hypselodoris tryoni	**PQ035999**		This study
	Ceratosoma	*Ceratosoma* sp.	OM135498		Unpublished
	Doriprismatica	*Doriprismatica atromarginata*	MN171300		Do et al. (2019)
	Verconia	*Verconia nivalis*	OL800586		Do et al.(2022)
	Glossodoris	*Glossodoris acosti*	MZ823409		Unpublished
	Goniobranchus	*Goniobranchus leopardus*	MZ747465		Unpublished
Cadlinidae	*Cadlina*	*Cadlina koreana*	MT919640		Do et al. (2022)
		Cadlina japonica	MT919639		Do et al. (2022)

				Cadlina umiushi	MT919641	Do et al. (2022)
			Aldisa	*Aldisa cooperi*	MT919638	Do et al. (2022)
		Actinocyclidae	*Actinocyclus*	***Actinocyclus* sp.**	**PQ035995**	This study
	Doridoidea	Dorididae	*Doris*	*Doris odhneri*	OL800585	Do et al. (2022)
		Discodorididae	*Carminodoris*	*Carminodoris armata*	OL800584	Do et al. (2022)
			Asteronotus	*Asteronotus hepaticus*	MW559976	Unpublished
			Halgerda	***Halgerda willeyi***	**PQ035992**	This study
	Polyceroidea	Polyceridae	*Triopha*	*Triopha modesta*	MW387958	Do et al. (2022)
			Polycera	*Polycera hedgpethi*	MZ713367	Unpublished
			Nembrotha	*Nembrotha kubaryana*	KY131978	Xiang et al. (2017)
			Tyrannodoris	*Tyrannodoris europaea*	AY083457	Grande et al. (2002)
	Onchidoridoidea	Aegiridae	*Notodoris*	*Notodoris gardineri*	DQ991934	Medina et al. (2011)
		Goniodorididae	*Ceratodoris*	*Ceratodoris hiroi*	MW408699	Do et al. (2021)
Cladobranchia	Tritonioidea	Tritoniidae	*Tritonia*	*Tritonia tetraquetra*	KP764765	Sevigny et al. (2015)
				Tritonia iocasica	OQ695495	Unpublished
			Tritoniopsis	*Tritoniopsis elegans*	OQ695496	Unpublished
	Arminoidea	Arminidae	*Dermatobranchus*	*Dermatobranchus otome*	MT527185	Do et al. (2020b)
				Armina variolosa	**PQ035993**	This study
	Dendronotoidea	Tethydidae	*Melibe*	*Melibe leonina*	KP764764	Sevigny et al. (2015)
	Aeolidioidea	Myrrhinidae	*Hermissenda*	*Hermissenda emurai*	MK279704	Dinh et al. (2019)
		Pleurolidiidae	*Protaeolidiella*	*Protaeolidiella atra*	MN911169	Do et al. (2020a)
		Aeolidiidae	*Berghia*	*Berghia stephanieae*	MW027646	Melo Clavijo J (2021)
			Spurilla	*Spurilla braziliana*	LC759638	Mizobata et al.(2023)
		Facelinidae	*Facelina*	*Facelina auriculata*	OP661154	Galià-Camps et al. (2024)
			Sakuraeolis	*Sakuraeolis japonica*	KX610997	Karagozlu et al. (2016b)
			Facelina	*Facelina bostoniensis*	OQ772261	Unpublished

		Sakuraeolis	***Sakuraeolis enosimensis***	**PQ035997**	This study
		Caloria	***Caloria militaris***	**PQ035994**	This study
Dendronotoidea	Dotidae	*Doto*	*Doto coronata*	OQ630514	Unpublished
	Dendronotidae	*Dendronotus*	*Dendronotus frondosus*	OQ626204	Unpublished
			Dendronotus primorjensis	**PQ035996**	This study
Fionoidea	Coryphellidae	*Coryphella*	*Coryphella verrucosa*	OQ596981	Unpublished
	Samlidae	*Samla*	***Samla bicolor***	**PQ035991**	This study
	Trinchesiidae	*Tenellia*	*Tenellia* sp.	ON553001	Unpublished
Aplysioidea	Aplysiidae	*Aplysia*	*Aplysia californica*	AY569552	Knudsen et al. (2006)
Aplysioidea	Aplysiidae	*Aplysia*	*Aplysia dactylomela*	DQ991927	Medina et al. (2011)

References

Dinh Do T, Kim JI, Jung DW, Choi TJ, Karagozlu MZ, Kim CB (2019) Characterization of the complete mitochondrial genome of *Herm issenda emurai* (Baba, 1937) (Nudibranchia, Facelinidae). Mitochondrial DNA Part B 4: 860-861. https://doi.org/10.1080/2380235 9.2019.1572477

Do TD, Choi TJ, Jung DW, An HE, Kim CB (2020a) The mitochondrial genome analysis of *Protaeolidiella atra* Baba, 1955 from Kore a. Mitochondrial DNA Part B 5: 1277–1278. https://doi.org/10.1080/23802359.2020.1731375

Do TD, Choi Y, Jung DW, Kim CB (2020b) Caution and curation for complete mitochondrial genome from next-generation sequencin g: A case study from *Dermatobranchus otome* (Gastropoda, Nudibranchia). Anim Syst Evol Divers 36: 336–346. https://doi.org/1 0.5635/ASED.2020.36.4.039

Do TD, Jung DW, Choi TJ, An HE, Kim CB (2021) Characterization of the complete mitochondrial genome of *Okenia hiroi* (Baba, 19 38) (Nudibranchia, Goniodorididae). Mitochondrial DNA Part B 6: 1124–1125. https://doi.org/10.1080/23802359.2021.1901627

Do TD, Jung DW, Kim CB (2022) Molecular phylogeny of selected dorid nudibranchs based on complete mitochondrial genome. Scie ntific Reports 12: 18797. https://doi.org/10.1038/s41598-022-23400-9

Do TD, Karagozlu MZ, Nguyen VQ, Kim CB(2019) Sequencing and analysis of the complete mitogenome of *Doriprismatica atromargi nata* (Cuvier, 1804). Mitochondrial DNA Part B 4: 2894–2895. https://doi.org/10.1080/23802359.2019.1660276

Epstein HE, Hallas JM, Johnson RF, Lopez A, Gosliner TM (2019) Reading between the lines: revealing cryptic species diversity and c olour patterns in *Hypselodoris* nudibranchs (Mollusca: Heterobranchia: Chromodorididae). Zoological Journal of the Linnean Socie ty 186: 116-189. https://doi.org/10.1093/zoolinnean/zly048

Galià-Camps C, Araujo AK, Carmona L, Martín-Hervás MDR, Pola M, Palero F, Cervera JL (2024a) New mitogenomes of *Runcinidae* a nd *Facelinidae*: two understudied heterobranch families (Mollusca: Gastropoda). Mitochondrial DNA Part B 9: 771–776. https://d oi.org/10.1080/23802359.2024.2363365

Galià-Camps C, Schell T, Enguídanos A, Pegueroles C, Arnedo MA, Ballesteros M Greve C(2024b) Jumping through hoops: Structural rearrangements and accelerated mutation rates on *Dendrodorididae* (Mollusca: Nudibranchia) mitogenomes rumble their evolutio n. Molecular Phylogenetics and Evolution 201: 108218. https://doi.org/10.1016/j.ympev.2024.108218

Grande C, Templado J, Lucas Cervera J, Zardoya R(2002) The complete mitochondrial genome of the nudibranch *Roboastra europaea* (Mollusca: Gastropoda) supports the monophyly of opisthobranchs. Molecular Biology and Evolution 19: 1672–1685. https://doi.org/10.1093/oxfordjournals.molbev.a003990

Johnson RF (2011) Breaking family ties: taxon sampling and molecular phylogeny of chromodorid nudibranchs (Mollusca, Gastropoda). Zoologica Scripta 40: 137-157. https://doi.org/10.1111/j.1463-6409.2010.00457.x

Johnson RF, Gosliner TM (2012) Traditional taxonomic groupings mask evolutionary history: a molecular phylogeny and new classification of the chromodorid nudibranchs. PLoS ONE 7: e33479. https://doi.org/10.1371/journal.pone.0033479

Karagozlu M Z, Sung J, Lee J, Kwak W, Kim C B (2016a) Complete sequences of mitochondrial genome of *Hypselodoris festiva* (A. Adams, 1861) (Mollusca, Gastropoda, Nudibranchia). Mitochondrial DNA Part B 1: 266–267. https://doi.org/10.1080/23802359.2016.1159933

Karagozlu MZ, Sung J M, Lee J H, Kim SG, Kim CB (2016b) Complete mitochondrial genome analysis of *Sakuraeolis japonica* (Baba, 1937) (Mollusca, Gastropoda, Nudibranchia). Mitochondrial DNA Part B 1: 720–721. https://doi.org/10.1080/23802359.2016.1229587

Kim H, Yoon M, Kim KY, Jung YH (2021) The complete mitochondrial genome of sea slug *Phyllidiopsis krempfi* Pruvot-Fol, 1957 (Nudibranchia, Phyllidiidae) from Pacific Ocean. Mitochondrial DNA Part B 6: 1523–1524. https://doi.org/10.1080/23802359.2020.1823898

Knudsen B, Kohn AB, Nahir B, Moroz LL (2006) Complete DNA sequence of the mitochondrial genome of the sea-slug, *Aplysia californica*: conservation of the gene order in Euthyneura. Molecular Phylogenetics and Evolution 38: 459–469. https://doi.org/10.1016/j.ympev.2005.08.017

Lee S, Bae S (2024) The complete mitochondrial genome of *Dendrodoris krusensternii* (Gastropoda, Nudibranchia, Dendrodorididae) from South Korea. Mitochondrial DNA Part B 9: 1636–1641.

Li Z, Zeng X, Ni G (2022) The complete mitochondrial genome of sea slug *Phyllidia elegans* Bergh, 1869 (Nudibranchia, Phyllidiidae) from the South China Sea. Mitochondrial DNA Part B 7: 1734–1736. https://doi.org/10.1080/23802359.2024.2435915

Lin GM, Xiang P, Audira G, Hsia C D (2019) Low coverage whole genome sequencing yields the complete mitogenome of *Hypselodoris bullockii* and *Hypselodoris apolegma* (Mollusca: Chromodorididae). J. Coast. Res. 97: 23–28. https://doi.org/10.2112/SI97-004.1

Lin GM, Xiang P, Sampurna BP, Hsiao CD (2017) Genome skimming yields the complete mitogenome of *Chromodoris annae* (Mollusca: Chromodorididae). Mitochondrial DNA Part B 2: 609–610. https://doi.org/10.1080/23802359.2017.1372715

Liu H, Shen P, Lin Q, Zhang H (2023) Complete mitochondrial genome of sea slug *Phyllidiella* sp. (Phyllidiidae, *Phyllidiella*) from the South China Sea and its phylogenetic implications. Research Square. https://doi.org/10.21203/rs.3.rs-2442215/v1

Liu H, Zhang H, Liu X, Lin Q, Shen PP (2024) Complete mitogenome data of sea slug *Phyllidiella pustulosa* nanhai (Phyllidiidae, *Phyllidiella*) from the South China Sea and its phylogenetic implications. SSRN.

Medina M, Lal S, Vallès Y, Takaoka TL, Dayrat BA, Boore JL, Gosliner T (2011) Crawling through time: transition of snails to slugs dating back to the Paleozoic, based on mitochondrial phylogenomics. Marine Genomics 4: 51-59. https://doi.org/10.1016/j.margen.2010.12.006

Melo Clavijo J, Drews F, Pirritano M, Simon M, Salhab A, Donath A, Christa G (2021) The complete mitochondrial genome of the photosymbiotic sea slug Berghia stephanieae (Valdés, 2005)(Gastropoda, Nudibranchia). Mitochondrial DNA Part B, *6*: 2281-2284. https://doi.org/10.1080/23802359.2021.1914211

Mizobata H, Hayashi K, Yonezawa R, Lanza A, Kinoshita S, Yoshitake K, Asakawa S (2023) The complete mitochondrial genome of *Spurilla braziliana* MacFarland 1909 (Nudibranchia, Aeolidiidae). Mitochondrial DNA Part B 8: 862–866. https://doi.org/10.1080/23802359.2023.2241693

Sevigny JL, Kirouac LE, Thomas WK, Ramsdell JS, Lawlor KE, Sharifi O, Newcomb JM (2015) The mitochondrial genomes of the nudibranch mollusks, *Melibe leonina* and *Tritonia diomedea*, and their impact on gastropod phylogeny. PLoS ONE 10: e0127519. https://doi.org/10.1371/journal.pone.0127519

Xiang P, Lin M, Wang Y, Shen KN, Hsiao CD (2016a) The complete mitogenome of sea slug, *Phyllidia ocellata* (Mollusca: Phyllidiidae). Mitochondrial DNA Part B 1: 96–97. https://doi.org/10.1080/23802359.2015.1137842

Xiang P, Lin M, Zhao L, Shen KN, Hsiao CD (2016b) Low-coverage genome sequencing yields the complete mitogenome of pyjamaslug, *Chromodoris quadricolor* (Mollusca: Chromodorididae). Mitochondrial DNA Part B 1: 94–95. https://doi.org/10.1080/23802359.2015.1137841

Xiang P, Lin M, Zhao L, Shen KN, Hsiao CD (2017) The complete mitogenome of sea slug, *Nembrotha kubaryana* (Mollusca: Polyceridae). Conserv. Genet. Resour. 9: 45–247. https://doi.org/10.1007/s12686-016-0663-3

Yu C, Kim H, Kim HJ, Jung YH (2018) The complete mitochondrial genome of the oriental sea slug: *Chromodoris orientalis* (Nudibranchia, Chromodorididae). Mitochondrial DNA Part B 3: 1017–1018. https://doi.org/10.1080/23802359.2018.1508381

Table S2. Structural features of Nudibranchia mitochondrial genomes.

Hypselodoris bullockii	Strand	Location	Size (bp)	Start Codon	Stop codon	Intergenic nucleotides
Cox1	H	1-1533	1533	ATG	TAA	10
tRNA-Val	H	1545-1609	65			0
16s	H	1610-2713	1104			0
tRNA-Leu	H	2714-2774	61			-5
tRNA-Ala	H	2775-2842	68			7
tRNA-Pro	H	2850-2917	68			1
Nad6	H	2918-3376	459	TTG	TAA	43
Nad5	H	3420-5042	1623	ATG	TAG	-20
Nad1	H	5023-5919	897	GTG	TAA	21
tRNA-Tyr	H	5943-5995	53			4
tRNA-Trp	H	6001-6066	66			0
Nad4L	H	6067-6360	294	TTG	TAA	-8
Cytb	H	6353-7480	1128	ATG	TAG	-6
tRNA-Asp	H	7476-7545	70			-1
tRNA-Phe	H	7546-7613	68			2
Cox2	H	7616-8290	675	ATG	TAA	3
tRNA-Gly	H	8295-8357	63			-1
tRNA-His	H	8358-8427	70			4
tRNA-Cys	H	8433-8497	65			208
tRNA-Ser	H	8708-8767	60			-1
Nad4	H	8768-10081	1314	ATG	TAA	93
tRNA-Gln	L	10176-10237	62			24
tRNA-Leu	L	10263-10324	62			3
Atp8	L	10328-10483	156	ATG	TAA	-1
tRNA-Asn	L	10484-10550	67			11
Atp6	L	10562-11236	675	ATA	TAG	2
tRNA-Arg	L	11240-11302	63			10
tRNA-Glu	L	11314-11378	65			0
12s	L	11379-12118	740			0
tRNA-Met	L	12119-12185	67			-2
Nad3	L	12184-12537	354	ATG	TAA	25
tRNA-Ser	L	12567-12618	52			29
tRNA-Thr	L	12658-12723	66			-5
Cox3	L	12719-13498	780	ATG	TAG	53
tRNA-Ile	H	13553-13616	64			0
Nad2	H	13617-14558	942	ATG	TAG	122
tRNA-Lys	H	14682-14749	68			1

Hypselodoris tryoni	Strand	Location	Size (bp)	Start Codon	Stop codon	Intergenic nucleotides
Cox1	H	1-1533	1533	ATG	TAA	14
tRNA-Val	H	1548-1613	66			0
16s	H	1614-2724	1111			0
tRNA-Leu	H	2725-2795	71			-5
tRNA-Ala	H	2791-2859	69			7
tRNA-Pro	H	2867-2933	67			1
Nad6	H	2935-3402	468	TTG	TAA	-14
Nad5	H	3389-5068	1680	ATG	TAG	-20
Nad1	H	5049-5960	912	GTG	TAG	10
tRNA-Tyr	H	5971-6025	55			4
tRNA-Trp	H	6030-6098	69			0
Nad4L	H	6099-6386	288	ATG	TAA	-1
Cytb	H	6386-7513	1128	ATG	TAG	-6
tRNA-Asp	H	7508-7574	67			-1
tRNA-Phe	H	7574-7642	69			3
Cox2	H	7646-8320	675	ATG	TAG	2
tRNA-Gly	H	8323-8389	67			-1
tRNA-His	H	8389-8460	72			4
tRNA-Cys	H	8465-8530	66			184
tRNA-Ser	H	8715-8777	63			-1
Nad4	H	8777-10090	1314	TTG	TAG	203
tRNA-Gln	L	10294-10357	64			18
tRNA-Leu	L	10376-10438	63			4
Atp8	L	10443-10598	156	ATG	TAG	-1
tRNA-Asn	L	10598-10671	74			19
Atp6	L	10691-11356	666	ATG	TAA	-1
tRNA-Arg	L	11356-11422	67			16
tRNA-Glu	L	11439-11505	67			0
12s	L	11506-12247	742			0
tRNA-Met	L	12248-12313	66			-2
Nad3	L	12312-12665	354	ATG	TAA	27
tRNA-Ser	L	12693-12749	57			34
tRNA-Thr	L	12784-12850	67			-5
Cox3	L	12846-13625	780	ATG	TAG	53
tRNA-Ile	H	13679-13744	66			0
Nad2	H	13745-14683	939	ATG	TAA	129
tRNA-Lys	H	14813-14879	67			1

***Actinocyclus* sp.**	Strand	Location	Size (bp)	Start Codon	Stop codon	Intergenic nucleotides
Cox1	H	1-1530	1530	ATG	TAG	87
tRNA-Val	H	1618-1681	64			0
16s	H	1682-2818	1137			0
tRNA-Leu	H	2819-2885	67			-5
tRNA-Ala	H	2881-2942	62			17
tRNA-Pro	H	2960-3024	65			0
Nad6	H	3025-3492	468	TTG	TAA	43
Nad5	H	3536-5158	1623	ATA	TAG	-20
Nad1	H	5139-6053	915	ATG	TAA	-2
tRNA-Tyr	H	6052-6115	64			-4
tRNA-Trp	H	6112-6179	68			0
Nad4L	H	6180-6473	294	GTG	TAA	-8
Cytb	H	6466-7593	1128	ATG	TAA	-6
tRNA-Asp	H	7588-7654	67			-1
tRNA-Phe	H	7654-7720	67			1
Cox2	H	7722-8396	675	ATG	TAA	1
tRNA-Gly	H	8398-8465	68			7
tRNA-His	H	8473-8541	69			7
tRNA-Cys	H	8549-8626	78			102
tRNA-Gln	L	8729-8789	61			3
tRNA-Leu	L	8793-8855	63			2
Atp8	L	8858-9013	156	ATG	TAA	-1
tRNA-Asn	L	9013-9081	69			10
Atp6	L	9092-9754	663	ATG	TAA	-1
tRNA-Arg	L	9754-9820	67			22
tRNA-Glu	L	9843-9913	71			0
12s	L	9914-10630	717			0
tRNA-Met	L	10631-10696	66			-2
Nad3	L	10695-11048	354	ATG	TAA	-35
tRNA-Ser	L	11014-11141	128			-13
tRNA-Ser	H	11129-11222	94			-22
Nad4	H	11201-12544	1344	GTG	TAG	6
tRNA-Thr	L	12551-12616	66			5
Cox3	L	12612-13391	780	ATG	TAG	53
tRNA-Ile	H	13445-13511	67			0
Nad2	H	13512-14447	936	ATG	TAA	3
tRNA-Lys	H	14515-14514	64			0

Halgerda willeyi	Strand	Location	Size (bp)	Start Codon	Stop codon	Intergenic nucleotides
Cox1	H	1-1533	1533	ATG	TAA	17
tRNA-Val	H	1551-1617	67			0
16s	H	1618-2732	1115			0
tRNA-Leu	H	2733-2800	68			-5
tRNA-Ala	H	2796-2861	66			4
tRNA-Pro	H	2866-2932	67			0
Nad6	H	2933-3400	468	TTG	TAA	43
Nad5	H	3444-5066	1623	ATA	TAG	-20
Nad1	H	5047-5958	912	GTG	TAA	11
tRNA-Tyr	H	5970-6025	56			3
tRNA-Trp	H	6029-6094	66			0
Nad4L	H	6095-6388	294	GTG	TAA	-8
Cytb	H	6381-7508	1128	ATG	TAA	-6
tRNA-Asp	H	7503-7571	69			-1
tRNA-Phe	H	7571-7638	68			1
Cox2	H	7640-8314	675	ATG	TAG	4
tRNA-Gly	H	8319-8387	69			7
tRNA-His	H	8395-8461	67			14
tRNA-Cys	H	8476-8540	65			89
tRNA-Gln	L	8630-8692	63			27
tRNA-Leu	L	8720-8784	65			5
Atp8	L	8790-8945	156	ATG	TAA	-1
tRNA-Asn	L	8945-9014	70			10
Atp6	L	9025-9702	678	ATG	TAA	2
tRNA-Arg	L	9705-9765	61			10
tRNA-Glu	L	9776-9843	68			0
12s	L	9844-10581	738			0
tRNA-Met	L	10582-10648	67			-2
Nad3	L	10647-11000	354	ATG	TAA	32
tRNA-Ser	L	11033-11091	59			4
tRNA-Ser	H	11096-11157	62			0
Nad4	H	11158-12483	1326	ATG	TAG	21
tRNA-Thr	L	12505-12569	65			-5
Cox3	L	12565-13344	780	ATG	TAG	53
tRNA-Ile	H	13398-13464	67			0
Nad2	H	13465-14397	933	ATG	TAG	-2
tRNA-Lys	H	14396-14459	64			0

Phyllidiella nigra	Strand	Location	Size (bp)	Start Codon	Stop codon	Intergenic nucleotides
Cox1	H	1-1530	1530	ATG	TAA	10
tRNA-Val	H	1541-1606	66			0
16s	H	1607-2747	1141			0
tRNA-Leu	H	2748-2815	68			-5
tRNA-Ala	H	2811-2879	69			9
tRNA-Pro	H	2889-2953	65			1
Nad6	H	2955-3425	471	TTG	TAA	43
Nad5	H	3469-5091	1623	ATG	TAG	-23
Nad1	H	5069-5983	915	ATG	TAA	6
tRNA-Tyr	H	5990-6055	66			1
tRNA-Trp	H	6057-6120	64			0
Nad4L	H	6121-6414	294	GTG	TAA	-8
Cytb	H	6407-7537	1131	ATG	TAG	-6
tRNA-Asp	H	7532-7601	70			1
tRNA-Phe	H	7603-7668	66			1
Cox2	H	7670-8347	678	ATG	TAA	7
tRNA-Gly	H	8355-8419	65			1
tRNA-His	H	8421-8484	64			11
tRNA-Cys	H	8496-8567	72			233
tRNA-Gln	L	8801-8863	63			7
tRNA-Leu	L	8871-8934	64			7
Atp8	L	8942-9097	156	ATG	TAA	0
tRNA-Asn	L	9098-9161	64			25
Atp6	L	9187-9855	669	GTG	TAA	-1
tRNA-Arg	L	9855-9920	66			22
tRNA-Glu	L	9943-10011	69			0
12s	L	10012-10754	743			0
tRNA-Met	L	10755-10822	68			-38
Nad3	L	10785-11174	390	GTG	TAA	45
tRNA-Ser	L	11220-11281	62			3
tRNA-Ser	H	11285-11347	63			1
Nad4	H	11349-12665	1317	ATG	TAA	29
tRNA-Thr	L	12695-12760	66			-5
Cox3	L	12756-13535	780	ATG	TAG	53
tRNA-Ile	H	13589-13661	73			1
Nad2	H	13663-14631	969	ATG	TAA	-36
tRNA-Lys	H	14596-14662	67			1

Phyllidia varicosa	Strand	Location	Size (bp)	Start Codon	Stop codon	Intergenic nucleotides
Cox1	H	1-1530	1530	ATG	TAA	12
tRNA-Val	H	1543-1610	68			0
16s	H	1611-2753	1143			0
tRNA-Leu	H	2754-2815	62			-5
tRNA-Ala	H	2811-2882	72			7
tRNA-Pro	H	2890-2954	65			1
Nad6	H	2956-3429	474	TTG	TAA	43
Nad5	H	3473-5095	1623	ATA	TAG	-23
Nad1	H	5073-5987	915	ATG	TAA	2
tRNA-Tyr	H	5990-6053	64			1
tRNA-Trp	H	6055-6123	69			0
Nad4L	H	6124-6417	294	GTG	TAA	-8
Cytb	H	6410-7537	1128	ATG	TAG	-6
tRNA-Asp	H	7532-7587	56			17
tRNA-Phe	H	7605-7671	67			1
Cox2	H	7673-8350	678	ATG	TAA	2
tRNA-Gly	H	8353-8416	64			4
tRNA-His	H	8421-8487	67			16
tRNA-Cys	H	8504-8572	69			162
tRNA-Gln	L	8735-8797	63			9
tRNA-Leu	L	8807-8873	67			2
Atp8	L	8876-9031	156	ATG	TAA	2
tRNA-Asn	L	9034-9100	67			11
Atp6	L	9112-9786	675	ATG	TTA	-1
tRNA-Arg	L	9786-9851	66			15
tRNA-Glu	L	9867-9933	67			0
12s	L	9934-10673	740			0
tRNA-Met	L	10674-10740	67			2
Nad3	L	10743-11096	354	ATG	TAA	27
tRNA-Ser	L	11124-11188	65			-11
tRNA-Ser	H	11178-11236	59			11
Nad4	H	11248-12594	1347	ATG	TAG	32
tRNA-Thr	L	12627-12693	67			-5
Cox3	L	12689-13468	780	ATG	TAG	53
tRNA-Ile	H	13522-13589	68			2
Nad2	H	13592-14545	954	ATG	TAG	-21
tRNA-Lys	H	14525-14591	67			1

Armina variolosa	Strand	Location	Size (bp)	Start Codon	Stop codon	Intergenic nucleotides
Cox1	H	1-1530	1530	GTG	TAA	11
tRNA-Val	H	1542-1608	67			0
16s	H	1609-2750	1142			0
tRNA-Leu	H	2751-2821	71			-8
tRNA-Ala	H	2814-2882	69			34
tRNA-Pro	H	2917-2980	64			2
Nad6	H	2983-3450	468	TTG	TAA	-14
Nad5	H	3437-5125	1689	ATG	TAA	31
Nad1	H	5095-6006	912	ATG	TAG	8
tRNA-Tyr	H	6015-6075	61			2
tRNA-Trp	H	6078-6140	63			0
Nad4L	H	6141-6434	294	TTG	TAA	-8
Cytb	H	6427-7560	1134	ATG	TAA	24
tRNA-Asp	H	7585-7651	67			4
tRNA-Phe	H	7656-7721	66			0
Cox2	H	7722-8396	675	ATG	TAA	1
tRNA-Gly	H	8398-8463	66			26
tRNA-His	H	8490-8553	64			4
tRNA-Cys	H	8558-8621	64			70
tRNA-Gln	L	8692-8753	62			-3
tRNA-Leu	L	8751-8813	63			-32
Atp8	L	8782-8967	186	ATG	TAA	3
tRNA-Asn	L	8971-9035	65			8
Atp6	L	9044-9706	663	ATG	TAA	2
tRNA-Arg	L	9709-9773	65			8
tRNA-Glu	L	9782-9846	65			0
12s	L	9847-10585	739			0
tRNA-Met	L	10586-10650	65			-2
Nad3	L	10649-11002	354	ATG	TAA	15
tRNA-Ser	L	11018-11097	80			55
tRNA-Ser	H	11153-11216	64			0
Nad4	H	11217-12530	1314	ATG	TAA	30
tRNA-Thr	L	12561-12626	66			-5
Cox3	L	12622-13401	780	ATG	TAA	52
tRNA-Ile	H	13454-13520	67			0
Nad2	H	13521-14462	942	ATG	TAG	-2
tRNA-Lys	H	14461-14527	67			0

Dendronotus primorjensis	Strand	Location	Size (bp)	Start Codon	Stop codon	Intergenic nucleotides
Cox1	H	1-1530	1530	ATG	TAG	12
tRNA-Val	H	1543-1608	66			0
16s	H	1609-2723	1115			3
tRNA-Leu	H	2727-2794	68			-5
tRNA-Ala	H	2790-2856	67			16
tRNA-Pro	H	2873-2936	64			2
Nad6	H	2939-3406	468	TTG	TAA	43
Nad5	H	3450-5072	1623	ATA	TAG	-23
Nad1	H	5050-5967	918	TTG	TAG	1
tRNA-Tyr	H	5969-6031	63			3
tRNA-Trp	H	6035-6101	67			0
Nad4L	H	6102-6395	294	TTG	TAA	8
Cytb	H	6388-7593	1206	ATG	TAA	5
tRNA-Asp	H	7599-7665	67			23
tRNA-Phe	H	7689-7755	67			1
Cox2	H	7757-8431	675	ATG	TAA	1
tRNA-Gly	H	8433-8500	68			9
tRNA-His	H	8510-8574	65			9
tRNA-Cys	H	8584-8645	62			174
tRNA-Gln	L	8820-8881	62			-3
tRNA-Leu	L	8879-8943	65			-26
Atp8	L	8918-9097	180	ATG	TAA	-1
tRNA-Asn	L	9097-9158	62			17
Atp6	L	9176-9841	666	ATG	TAG	-1
tRNA-Arg	L	9841-9905	65			5
tRNA-Glu	L	9911-9977	67			0
12s	L	9978-10710	733			0
tRNA-Met	L	10711-10779	69			-1
Nad3	L	10779-11130	408	ATG	T--	26
tRNA-Ser	L	11157-11226	70			58
tRNA-Ser	H	11285-11342	58			1
Nad4	H	11344-12657	1314	ATG	TAA	23
tRNA-Thr	L	12681-12744	64			-5
Cox3	L	12740-13519	780	ATG	TAA	52
tRNA-Ile	H	13572-13640	69			1
Nad2	H	13642-14577	936	ATG	TAG	-1
tRNA-Lys	H	14577-14643	67			3

Samla bicolor	Strand	Location	Size (bp)	Start Codon	Stop codon	Intergenic nucleotides
Cox1	H	1-1533	1533	ATG	TAA	4
tRNA-Val	H	1538-1601	64			0
16s	H	1602-2689	1088			0
tRNA-Leu	H	2690-2755	66			-5
tRNA-Ala	H	2751-2815	65			-25
tRNA-Pro	H	2841-2904	64			2
Nad6	H	2907-3374	468	TTG	TAA	43
Nad5	H	3418-5040	1623	ATA	TAG	-23
Nad1	H	5018-5932	915	GTG	TAA	3
tRNA-Tyr	H	5936-5996	61			6
tRNA-Trp	H	6003-6071	69			0
Nad4L	H	6072-6365	294	TTG	TAA	-8
Cytb	H	6358-7479	1122	ATG	TAA	7
tRNA-Asp	H	7487-7555	69			-1
tRNA-Phe	H	7555-7621	67			1
Cox2	H	7623-8297	675	ATG	TAA	-1
tRNA-Gly	H	8297-8363	67			6
tRNA-His	H	8370-8432	63			0
tRNA-Cys	H	8433-8498	66			444
tRNA-Gln	L	8943-9000	58			-1
tRNA-Leu	L	9000-9063	64			2
Atp8	L	9066-9218	153	ATG	TAA	0
tRNA-Asn	L	9219-9284	66			2
Atp6	L	9287-9949	663	ATG	TAA	-1
tRNA-Arg	L	9949-10012	64			0
tRNA-Glu	L	10013-10077	65			0
12s	L	10078-10815	738			0
tRNA-Met	L	10816-10882	67			-23
Nad3	L	10860-11234	375	GTG	TAA	23
tRNA-Ser	L	11258-11334	77			48
tRNA-Ser	H	11383-11443	61			2
Nad4	H	11446-12759	1314	ATG	TAA	18
tRNA-Thr	L	12778-12843	66			-5
Cox3	L	12839-13618	780	ATG	TAA	51
tRNA-Ile	H	13670-13741	72			0
Nad2	H	13742-14677	936	ATG	TAG	0
tRNA-Lys	H	14678-14743	66			0

Sakuraeolis enosimensis	Strand	Location	Size (bp)	Start Codon	Stop codon	Intergenic nucleotides
Cox1	H	1-1548	1548	GTG	TAA	191
tRNA-Val	H	1740-1804	65			0
16s	H	1805-2858	1054			18
tRNA-Leu	H	2877-2941	65			-5
tRNA-Ala	H	2937-3001	65			3
tRNA-Pro	H	3005-3069	65			-14
Nad6	H	3056-3541	486	ATG	TAA	43
Nad5	H	3585-5207	1623	ATA	TAG	-23
Nad1	H	5185-6096	912	ATG	TAA	1
tRNA-Tyr	H	6098-6161	64			8
tRNA-Trp	H	6170-6235	66			0
Nad4L	H	6236-6523	288	TTG	TAA	-1
Cytb	H	6523-7647	1125	ATG	TAA	-6
tRNA-Asp	H	7642-7708	67			-1
tRNA-Phe	H	7708-7773	66			1
Cox2	H	7775-8467	693	ATG	TAA	-18
tRNA-Gly	H	8450-8516	67			6
tRNA-His	H	8523-8585	63			-3
tRNA-Cys	H	8583-8645	63			139
tRNA-Gln	L	8785-8848	64			-3
tRNA-Leu	L	8846-8909	64			-32
Atp8	L	8878-9063	186	ATG	TAA	1
tRNA-Asn	L	9065-9126	62			-26
Atp6	L	9101-9790	690	ATG	TTA	0
tRNA-Arg	L	9791-9854	64			12
tRNA-Glu	L	9867-9932	66			0
12s	L	9933-10632	700			0
tRNA-Met	L	10633-10701	69			-23
Nad3	L	10679-11053	375	TTG	TAA	24
tRNA-Ser	L	11078-11137	60			1
tRNA-Ser	H	11139-11197	59			0
Nad4	H	11198-12511	1314	ATG	TAA	30
tRNA-Thr	L	12542-12606	65			-5
Cox3	L	12602-13381	780	ATG	TAA	52
tRNA-Ile	H	13434-13498	65			0
Nad2	H	13499-14443	945	GTG	TAG	-2
tRNA-Lys	H	14442-14505	64			1

Caloria militaris	Strand	Location	Size (bp)	Start Codon	Stop codon	Intergenic nucleotides
Cox1	H	1-1530	1530	GTG	TAA	7
tRNA-Val	H	1538-1603	66			0
16s	H	1604-2686	1083			0
tRNA-Leu	H	2687-2751	65			0
tRNA-Ala	H	2752-2807	56			9
tRNA-Pro	H	2817-2880	64			1
Nad6	H	2882-3352	471	TTG	TAA	43
Nad5	H	3396-5018	1623	ATG	TAG	23
Nad1	H	4996-5913	918	TTG	TAG	4
tRNA-Tyr	H	5918-5983	66			-2
tRNA-Trp	H	5982-6048	67			0
Nad4L	H	6049-6342	294	TTG	TAA	-8
Cytb	H	6335-7456	1122	ATG	TAG	0
tRNA-Asp	H	7457-7524	68			-1
tRNA-Phe	H	7524-7589	66			1
Cox2	H	7591-8266	676	ATG	T--	-1
tRNA-Gly	H	8266-8331	66			0
tRNA-His	H	8332-8390	59			0
tRNA-Cys	H	8391-8455	65			116
tRNA-Gln	L	8572-8635	64			-3
tRNA-Leu	L	8633-8698	66			-32
Atp8	L	8667-8852	186	ATG	TAA	0
tRNA-Asn	L	8853-8914	62			12
Atp6	L	8927-9589	663	ATG	TAA	-1
tRNA-Arg	L	9589-9652	64			6
tRNA-Glu	L	9659-9722	64			0
12s	L	9723-10430	708			0
tRNA-Met	L	10431-10495	65			-2
Nad3	L	10494-10847	354	TTG	TAG	26
tRNA-Ser	L	10874-10935	62			-3
tRNA-Ser	H	10933-10990	58			1
Nad4	H	10992-12377	1386	GTG	TAG	-41
tRNA-Thr	L	12337-12401	65			-5
Cox3	L	12397-13176	780	ATG	TAA	52
tRNA-Ile	H	13229-13295	67			2
Nad2	H	13298-14236	939	ATG	TAA	-3
tRNA-Lys	H	14234-14298	65			1

Table S3. AT and GC Skew rate of Nudibranchia mitochondrial genomes.

Hypselodoris bullockii	T	C	A	G	TOTAL	(A+T)%	AT skew	GC skew
Whole genome	38.5	13.4	30.8	17.2	14750	69.3	-0.111	0.124
PCGs	41	14	27.6	17.4	10830	68.6	-0.195	0.108
PCGs1	33.4	14.3	28.4	23.9	3610	61.8	-0.081	0.251
PCGs2	45.3	19.2	17.8	17.8	3610	63.1	-0.436	-0.038
PCGs3	44.4	8.4	36.7	10.5	3610	81.1	-0.095	0.111
ATP6	37.3	18.7	30.1	13.9	675	67.4	-0.107	-0.147
ATP8	36.5	14.7	36.5	12.2	156	73	0.000	-0.093
COX1	41.7	15.6	23.4	19.3	1533	65.1	-0.281	0.106
COX2	35.7	14.8	31.1	18.4	675	66.8	-0.069	0.108
COX3	37.8	17.6	26.5	18.1	780	64.3	-0.176	0.014
CYTB	40.5	15.2	28.5	15.7	1128	69	-0.174	0.016
NAD1	42.4	14.6	25.8	17.3	897	68.2	-0.243	0.085
NAD2	41.7	10.5	27.2	20.6	942	68.9	-0.210	0.325
NAD3	42.9	15.8	26.6	14.7	354	69.5	-0.235	-0.036
NAD4	42.9	11.9	28.3	16.8	1314	71.2	-0.205	0.171
NAD4L	45.6	13.9	24.5	16	294	70.1	-0.301	0.070
NAD5	41.3	11.6	30.4	16.6	1623	71.7	-0.152	0.177
NAD6	45.3	9.6	25.1	20	459	70.4	-0.287	0.351
TRNA	34	13.6	35	17.4	1455	69	0.014	0.123
16SRNA	36.3	11.3	35.7	16.7	1100	72	-0.008	0.193
12SRNA	31.7	15	39.2	14.1	739	70.9	0.106	-0.031

Hypselodoris tryoni	T	C	A	G	TOTAL	(A+T)%	AT skew	GC skew
Whole genome	36.5	16.3	29.3	17.8	14880	65.8	-0.109	0.044
PCGs	38.6	17.1	25.8	18.5	10893	64.4	-0.199	0.039
PCGs1	32.4	15.9	27.5	24.2	3631	59.9	-0.082	0.207
PCGs2	44.7	20.3	17.6	17.4	3631	62.3	-0.435	-0.077
PCGs3	38.9	15.1	32.1	13.9	3631	71	-0.096	-0.041
Atp6	38.7	16.7	27.6	17	666	66.3	-0.167	0.009
Atp8	36.5	13.5	37.2	12.8	156	73.7	0.009	-0.027
Cox1	38.6	18.5	23.4	19.6	1533	62	-0.245	0.029
Cox2	34.5	16.7	28.6	20.1	675	63.1	-0.094	0.092
Cox3	37.2	18.6	26.3	17.9	780	63.5	-0.172	-0.019
Cytb	37.4	19.3	25.3	18	1128	62.7	-0.193	-0.035
Nad1	39.9	18	24.5	17.7	912	64.4	-0.239	-0.008
Nad2	39.1	14.6	24.4	21.9	939	63.5	-0.231	0.200
Nad3	41.8	16.4	25.7	16.1	354	67.5	-0.239	-0.009
Nad4	39.3	16.9	25.3	18.5	1314	64.6	-0.217	0.045
Nad4L	40.6	20.1	23.6	15.6	288	64.2	-0.265	-0.126
Nad5	38.5	15.6	27.7	18.2	1680	66.2	-0.163	0.077
Nad6	42.5	14.5	24.6	18.4	468	67.1	-0.267	0.119
TRNA	34.1	14.7	33.2	18	1459	67.3	-0.013	0.101
16SRNA	34.8	12.2	36.5	16.4	1111	71.3	0.024	0.147
12SRNA	31.7	14.7	38.4	15.2	742	70.1	0.096	0.017

***Actinocyclus* sp.**	T	C	A	G	TOTAL	(A+T)%	AT skew	GC skew
Whole genome	38.9	14.1	28	19	14514	66.9	-0.163	0.148
PCGs	41.2	14.8	25.2	18.8	10866	66.4	-0.241	0.119
PCGs1	33.3	14.8	26.7	25.2	3622	60	-0.110	0.260
PCGs2	44.8	20.1	17.5	17.6	3622	62.3	-0.438	-0.066
PCGs3	45.5	9.6	31.4	13.5	3622	76.9	-0.183	0.169
Atp6	36.7	20.1	26.5	16.7	663	63.2	-0.161	-0.092
Atp8	41.7	16.7	31.4	10.3	156	73.1	-0.141	-0.237
Cox1	41.7	15.7	23.5	19.1	1530	65.2	-0.279	0.098
Cox2	35.6	16.3	28.3	19.9	675	63.9	-0.114	0.099
Cox3	34.2	21.9	26.8	17.1	780	61	-0.121	-0.123
Cytb	40.1	16.6	25.1	18.3	1128	65.2	-0.230	0.049
Nad1	42.1	13.2	24	20.7	915	66.1	-0.274	0.221
Nad2	44.3	11.2	22.9	21.6	936	67.2	-0.318	0.317
Nad3	39	16.4	29.9	14.7	354	68.9	-0.132	-0.055
Nad4	42.3	12.9	24.6	20.2	1344	66.9	-0.265	0.221
Nad4L	46.3	13.6	22.1	18	294	68.4	-0.354	0.139
Nad5	43.1	12.9	26.1	17.9	1623	69.2	-0.246	0.162
Nad6	49.6	8.1	23.3	19	468	72.9	-0.361	0.402
TRNA	34.5	15.1	30.8	19.5	1555	65.3	-0.057	0.127
16SRNA	37.1	11.8	33.1	18	1137	70.2	-0.057	0.208
12SRNA	28.5	15.5	39.3	16.7	717	67.8	0.159	0.037

Halgerda willeyi	T	C	A	G	TOTAL	(A+T)%	AT skew	GC skew
Whole genome	34.9	16.5	33.2	15.4	14459	68.1	-0.025	-0.034
PCGs	38.1	16.5	29.3	16	10860	67.4	-0.131	-0.015
PCGs1	31.2	16.2	28.2	24.3	3620	59.4	-0.051	0.200
PCGs2	44.4	21.6	17.6	16.5	3620	62	-0.432	-0.134
PCGs3	38.8	11.7	42.2	7.3	3620	81	0.042	-0.232
Atp6	40.9	15.9	27.3	15.9	678	68.2	-0.199	0.000
Atp8	41	12.2	31.4	15.4	156	72.4	-0.133	0.116
Cox1	36.2	18.5	28.2	17.1	1533	64.4	-0.124	-0.039
Cox2	30.8	17	33.6	18.5	675	64.4	0.043	0.042
Cox3	42.9	15.1	22.7	19.2	780	65.6	-0.308	0.120
Cytb	33.8	20.8	29.9	15.5	1128	63.7	-0.061	-0.146
Nad1	37.2	18.1	28.5	16.2	912	65.7	-0.132	-0.055
Nad2	37.9	15.6	29.7	16.7	933	67.6	-0.121	0.034
Nad3	44.4	12.4	26.3	16.9	354	70.7	-0.256	0.154
Nad4	40.6	15.3	30.3	13.7	1326	70.9	-0.145	-0.055
Nad4L	38.8	18.4	28.9	13.9	294	67.7	-0.146	-0.139
Nad5	37.5	15.2	32.7	14.6	1623	70.2	-0.068	-0.020
Nad6	44.4	11.3	28.2	16	468	72.6	-0.223	0.172
TRNA	33.7	13.8	33.9	18.6	1439	67.6	0.003	0.148
16SRNA	33	13.4	38.7	15	1115	71.7	0.079	0.056
12SRNA	32.1	13	38.3	16.5	738	70.4	0.088	0.119

Phyllidiella nigra	T	C	A	G	TOTAL	(A+T)%	AT skew	GC skew
Whole genome	37.6	14.6	30.9	16.8	14663	68.5	-0.098	0.070
PCGs	40.6	14.9	27.3	17.2	10923	67.9	-0.196	0.072
PCGs1	34.3	13.9	27.3	24.5	3641	61.6	-0.114	0.276
PCGs2	44.5	21.1	17.5	16.9	3641	62	-0.435	-0.111
PCGs3	43.1	9.7	37	10.2	3641	80.1	-0.076	0.025
Atp6	39.5	17.9	27.2	15.4	669	66.7	-0.184	-0.075
Atp8	37.8	17.3	33.3	11.5	156	71.1	-0.063	-0.201
Cox1	41.4	15	25.4	18.1	1530	66.8	-0.240	0.094
Cox2	34.7	16.1	30.5	18.7	678	65.2	-0.064	0.075
Cox3	38.5	16.4	26.5	18.6	780	65	-0.185	0.063
Cytb	38.1	17.1	27.6	17.2	1131	65.7	-0.160	0.003
Nad1	40.7	15.4	28.1	15.8	915	68.8	-0.183	0.013
Nad2	45.6	12.6	25.5	16.3	969	71.1	-0.283	0.128
Nad3	42.6	14.4	25.6	17.4	390	68.2	-0.249	0.094
Nad4	40.4	14	28.5	17.1	1317	68.9	-0.173	0.100
Nad4L	41.8	15.3	25.9	17	294	67.7	-0.235	0.053
Nad5	41.3	13.4	27.6	17.7	1623	68.9	-0.199	0.138
Nad6	44.8	11.5	26.5	17.2	471	71.3	-0.257	0.199
TRNA	34.1	13.5	34	18.4	1460	68.1	-0.001	0.154
16SRNA	35.1	12.2	35.8	16.9	1141	70.9	0.010	0.162
12SRNA	32.7	13.1	38.6	15.6	743	71.3	0.083	0.087

Phyllidia varicosa	T	C	A	G	TOTAL	(A+T)%	AT skew	GC skew
Whole genome	38.9	13.4	31.7	16	14592	70.6	-0.102	0.088
PCGs	42.1	13.8	27.4	16.6	10908	69.5	-0.212	0.092
PCGs1	35.1	13.2	28.2	23.5	3636	63.3	-0.109	0.281
PCGs2	45.2	20.4	17.9	16.5	3636	63.1	-0.433	-0.106
PCGs3	46.1	8	36.2	9.7	3636	82.3	-0.120	0.096
Atp6	41.5	16.3	28.7	13.5	675	70.2	-0.182	-0.094
Atp8	44.9	14.1	32.7	8.3	156	77.6	-0.157	-0.259
Cox1	41.6	15.6	24.8	18	1530	66.4	-0.253	0.071
Cox2	35.4	15.2	29.9	19.5	678	65.3	-0.084	0.124
Cox3	39.9	17.2	24.7	18.2	780	64.6	-0.235	0.028
Cytb	40.8	15	27.8	16.4	1128	68.6	-0.190	0.045
Nad1	42.4	13.2	26.9	17.5	915	69.3	-0.224	0.140
Nad2	46.9	11.3	25.1	16.8	954	72	-0.303	0.196
Nad3	47.2	13	25.1	14.7	354	72.3	-0.306	0.061
Nad4	42.1	13.2	28.4	16.3	1347	70.5	-0.194	0.105
Nad4L	6.9	11.9	26.9	14.3	294	33.8	0.592	0.092
Nad5	42.1	12.3	29.9	15.7	1623	72	-0.169	0.121
Nad6	44.3	9.7	28.7	17.3	474	73	-0.214	0.281
TRNA	34.9	12.2	36.4	16.5	1446	71.3	0.021	0.150
16SRNA	36.4	10.7	37.9	15	1143	74.3	0.020	0.167
12SRNA	32	11.9	41.5	14.6	740	73.5	0.129	0.102

Armina variolosa	T	C	A	G	TOTAL	(A+T)%	AT skew	GC skew
Whole genome	40.9	11.9	27.9	19.3	14527	68.8	-0.189	0.237
PCGs	43.2	12.7	24.9	19.2	10941	68.1	-0.269	0.204
PCGs1	35.6	12.2	27.1	25.1	3647	62.7	-0.136	0.346
PCGs2	46.3	18.8	17.1	17.9	3647	63.4	-0.461	-0.025
PCGs3	47.6	7.2	30.6	14.6	3647	78.2	-0.217	0.339
Atp6	36.3	19.3	30.2	14.2	663	66.5	-0.092	-0.152
Atp8	38.2	11.3	41.4	9.1	186	79.6	0.040	-0.108
Cox1	43.8	13.1	21.8	21.3	1530	65.6	-0.335	0.238
Cox2	39.1	12.9	25.3	22.7	675	64.4	-0.214	0.275
Cox3	35.5	19.1	29.5	15.9	780	65	-0.092	-0.091
Cytb	42.8	13.8	23.9	19.5	1134	66.7	-0.283	0.171
Nad1	44	12.3	24	19.7	912	68	-0.294	0.231
Nad2	46.4	10	23	20.6	942	69.4	-0.337	0.346

Nad3	40. 1	18.4	28.5	13	354	68.6	-0.169	-0.172
Nad4	45. 2	10.7	25.3	18.9	1314	70.5	-0.282	0.277
Nad4L	46. 3	8.8	23.5	21.4	294	69.8	-0.327	0.417
Nad5	46. 4	10.7	23.4	19.5	1689	69.8	-0.330	0.291
Nad6	47. 4	6.4	24.1	22	468	71.5	-0.326	0.549
TRNA	36. 5	12.3	33.9	17.3	1517	70.4	-0.037	0.169
16SRNA	38. 5	9.7	33.3	18.5	1142	71.8	-0.072	0.312
12SRNA	28. 7	16.2	41	14.1	739	69.7	0.176	-0.069

Dendronotus primorjensis	T	C	A	G	TOTAL	(A+T)%	AT skew	GC skew
Whole genome	37.4	15.1	26.8	20.6	14646	64.2	-0.165	0.154
PCGs	40.4	15.7	23.4	20.5	10941	63.8	-0.266	0.133
PCGs1	32.2	16	24.8	27	3647	57	-0.130	0.256
PCGs2	44.3	20.2	16.9	18.6	3647	61.2	-0.448	-0.041
PCGs3	44.7	11	28.3	16	3647	73	-0.225	0.185
Atp6	36.3	22.2	25.1	16.4	666	61.4	-0.182	-0.150
Atp8	38.9	18.9	31.1	11.1	180	70	-0.111	-0.260
Cox1	39.3	16.8	22	22	1530	61.3	-0.282	0.134
Cox2	34.8	15.9	26.4	23	675	61.2	-0.137	0.183
Cox3	36.3	21	24.4	18.3	780	60.7	-0.196	-0.069
Cytb	38.1	17.2	23.9	20.8	1206	62	-0.229	0.095
Nad1	43.5	14.5	21.2	20.8	918	64.7	-0.345	0.178
Nad2	42.3	12.8	22.	22.	936	64.8	-0.306	0.271

			5	3				
Nad3	40.7	18.5	22.8	17.9	351	63.5	-0.282	-0.016
Nad4	42.2	13.2	24.2	20.4	1314	66.4	-0.271	0.214
Nad4L	44.6	12.9	20.1	22.4	294	64.7	-0.379	0.269
Nad5	42.6	13.4	23.4	20.6	1623	66	-0.291	0.212
Nad6	45.9	11.1	20.9	22	468	66.8	-0.374	0.329
TRNA	34.5	14.7	30.9	19.9	1442	65.4	-0.055	0.150
16SRNA	34.5	12.9	32.2	20.4	1115	66.7	-0.034	0.225
12SRNA	26.5	18	38.5	17.1	733	65	0.185	-0.026

Samla bicolor	T	C	A	G	TOTAL	(A+T)%	AT skew	GC skew
Whole genome	36.7	16.2	31.7	15.4	14743	68.4	-0.158	-0.025
PCGs	40.3	15.5	27.7	16.5	10851	68	-0.451	0.031
PCGs1	33.1	14.8	29.1	23.1	3617	62.2	-0.138	0.219
PCGs2	45	20.5	17.6	16.9	3617	62.6	-1.564	-0.096
PCGs3	2.8	11.2	36.5	9.5	3617	39.3	0.923	-0.082
Atp6	42.5	14.9	26.7	15.8	663	69.2	-0.588	0.029
Atp8	41.2	14.4	31.4	13.1	153	72.6	-0.312	-0.047
Cox1	38.2	17.7	26	18.1	1533	64.2	-0.467	0.011
Cox2	35.9	16.9	28.9	18.4	675	64.8	-0.243	0.042
Cox3	39.7	14	25.9	20.4	780	65.6	-0.532	0.186
Cytb	38.9	19.3	25.8	15.9	1122	64.7	-0.507	-0.097
Nad1	39.3	16.5	26.2	17.9	915	65.5	-0.497	0.041
Nad2	41.2	14.4	29.1	15.3	936	70.3	-0.414	0.030
Nad3	45.	11.2	27.5	16	375	72.8	-0.647	0.176

	3							
Nad4	42.1	14.1	29.4	14.5	1314	71.5	-0.429	0.014
Nad4L	43.2	11.6	29.9	15.3	294	73.1	-0.446	0.138
Nad5	40.4	14.7	29.6	15.3	1623	70	-0.364	0.020
Nad6	42.7	13.5	27.1	16.7	468	69.8	-0.575	0.106
TRNA	35.3	13.2	34.9	16.6	1511	70.2	-0.011	0.114
16SRNA	35.6	12.4	36.7	15.3	1088	72.3	0.030	0.105
12SRNA	31	13.3	39.2	16.5	738	70.2	0.209	0.107

Sakuraeolis enosimensis	T	C	A	G	TOTAL	(A+T)%	AT skew	GC skew
Whole genome	38	14.6	28.4	19	14506	66.4	-0.145	0.131
PCGs	40.7	14.7	25.6	19	10965	66.3	-0.228	0.128
PCGs1	32.7	14.2	27.2	25.9	3655	59.9	-0.092	0.292
PCGs2	44.4	20.4	16.9	18.3	3655	61.3	-0.449	-0.054
PCGs3	44.9	9.6	32.8	12.7	3655	77.7	-0.156	0.139
Atp6	38.1	17.7	27.8	16.4	690	65.9	-0.156	-0.038
Atp8	34.9	14.5	36	14.5	186	70.9	0.016	0.000
Cox1	40.4	15.6	24.5	19.6	1548	64.9	-0.245	0.114
Cox2	35.5	15	28.9	20.6	693	64.4	-0.102	0.157
Cox3	36.7	18.6	25.9	18.8	780	62.6	-0.173	0.005
Cytb	40	15.9	25.7	18.4	1125	65.7	-0.218	0.073
Nad1	42.4	13.9	24.1	19.5	912	66.5	-0.275	0.168
Nad2	42.3	12.1	25.7	19.9	945	68	-0.244	0.244
Nad3	39.7	18.4	25.6	16.3	375	65.3	-0.216	-0.061
Nad4	42.4	13.7	25.3	18.6	1314	67.7	-0.253	0.152
Nad4L	46.2	10.4	24.3	19.1	288	70.5	-0.311	0.295
Nad5	41.9	13.9	24.6	19.7	1623	66.5	-0.260	0.173
Nad6	45.5	10.9	24.5	19.1	486	70	-0.300	0.273
TRNA	33.2	15.2	31.2	20.3	1418	64.4	-0.031	0.144
16SRNA	34.6	12.4	32.5	20.4	1054	67.1	-0.031	0.244
12SRNA	28.4	16.7	37.6	17.3	700	66	0.139	0.018

Caloria militaris	T	C	A	G	TOTAL	(A+T)%	AT skew	GC skew
Whole genome	37.6	15.2	27.6	19.6	14299	65.2	-0.153	0.126
PCGs	39.9	15.5	25.1	19.6	10941	65	-0.228	0.117
PCGs1	33.1	14.3	27.1	25.5	3647	60.2	-0.100	0.281
PCGs2	44.4	20.7	17.1	17.9	3647	61.5	-0.444	-0.073
PCGs3	42.3	11.4	31	15.4	3647	73.3	-0.154	0.149
Atp6	34.8	19.2	29.3	16.7	663	64.1	-0.086	-0.070
Atp8	32.3	16.7	36.6	14.5	186	68.9	0.062	-0.071
Cox1	39.9	15.6	24.3	20.1	1530	64.2	-0.243	0.126
Cox2	36.7	14.1	26.7	22.5	675	63.4	-0.158	0.230
Cox3	35.6	19.9	25.5	19	780	61.1	-0.165	-0.023
Cytb	39.3	18.6	23.1	19	1122	62.4	-0.260	0.011
Nad1	41.8	13.8	23.6	20.7	918	65.4	-0.278	0.200
Nad2	40.1	12.9	25.8	21.2	939	65.9	-0.217	0.243
Nad3	38.4	16.7	26.3	18.6	354	64.7	-0.187	0.054
Nad4	42.3	14.5	24.7	18.5	1386	67	-0.263	0.121
Nad4L	42.9	12.9	25.5	18.7	294	68.4	-0.254	0.184
Nad5	41.8	14.5	24.3	19.4	1623	66.1	-0.265	0.145
Nad6	44.4	11.7	22.5	21.4	471	66.9	-0.327	0.293
TRNA	32.8	14.8	32.8	19.7	1410	65.6	0.000	0.142
16SRNA	33.7	13.3	33.1	19.9	1083	66.8	-0.009	0.199
12SRNA	28.1	17.7	36.4	17.8	708	64.5	0.129	0.003

Table S4. Best fit partitions and substitution models.

Dataset	Set Partition	Best Model for BI	Best Model for ML
Best Partition CDS	*atp6-8* 1th	GTR+I+G	TIM2+F+I+G4
(BIC= 432,312.41)	*atp6-8* 2th	GTR+I+G	GTR+F+I+G4
	atp6-8 3th	HKY+I+G	TPM2+F+I+G4
	cox1-2-3 1th	GTR+I+G	GTR+F+I+G4
	cox1-2-3 2th	GTR+I+G	GTR+F+I+G4
	cox1-2-3 3th	HKY+I+G	HKY+F+I+G4
	cob 1th	TRN+I+G	TIM2+F+I+G4
	cob 2th	TVM+I+G	TVM+F+I+G4
	cob 3th	HKY+I+G	HKY+F+I+G4
	nad1-2-3-4-4L-5-6 1th	GTR+I+G	GTR+F+I+G4
	nad1-2-3-4-4L-5-6 2th	GTR+I+G	GTR+F+I+G4
	nad1-2-3-4-4L-5-6 3th	HKY+I+G	HKY+F+I+G4
Best Partition rRNAs (BIC = 32,958.35)	*rrnS-L*	GTR+I+G	GTR+F+I+G4

Table S5. Genetic distance of *Hypselodoris bullockii* cox1 in NCBI

	1	2	3	4	5	6	7	8	9
1.PQ035990									
2.**MF785092**	**0.1275**								
3.EU982743	0.0070	**0.1316**							
4.JQ727888	0.0028	**0.1289**	0.0081						
5.MG645568	0.0009	**0.1289**	0.0061	0.0018					
6.MG645569	0.0056	**0.1289**	0.0030	0.0063	0.0045				
7.MG645570	0.0018	**0.1275**	0.0071	0.0027	0.0009	0.0054			
8.MG645571	0.0056	**0.1289**	0.0030	0.0063	0.0045	0.0000	0.0054		
9.MG645572	0.0018	**0.1302**	0.0061	0.0027	0.0009	0.0054	0.0018	0.0054	

Table S6. Genetic distance of *Actinocyclus cox1* in NCBI

	1	2	3	4	5	6
1. ***Actinocyclus* sp. (PQ035995)**						
2. *Actinocyclus* cf. *verrucosus* (OQ573576)	**0.0013**					
3. *Actinocyclus verrucosus* (MF958438)	0.0771	0.0781				
4. *Actinocyclus verrucosus* (EF535108)	0.0735	0.0745	0.0021			
5. *Actinocyclus verrucosus* (MW277700)	0.0699	0.0708	0.0014	0.0020		
6. *Actinocyclus verrucosus* (MW278846)	0.0727	0.0736	0.0042	0.0034	0.0039	

Table S7. Genetic distances of *Armina cox1* in NCBI

	1	2	3	4	5	6	7	8	9	10	11	12	13	14	15	16	17
1. *Armina lovenii* (PQ739884)																	
2. *Armina variolosa* (PQ035993)	0.282																
3. *Armina* cf. *babai* (MW940365)	0.302	0.233															
4. *Armina gilchristi* (MW940362)	0.207	0.221	0.219														
5. *Armina* sp. C (MW940361)	0.311	0.248	0.244	0.243													
6. *Armina* sp. D (MW940353)	0.311	0.244	0.243	0.242	0.004												
7. *Armina californica* (MW940352)	0.275	0.272	0.307	0.263	0.278	0.285											
8. *Armina occulta* (MW940346)	0.303	0.261	0.265	0.273	0.291	0.292	0.218										
9. *Armina* sp. 9 (MW940342)	0.361	0.295	0.311	0.282	0.292	0.284	0.332	0.300									
10. ***Armina variolosa* (MW940328)**	0.285	**0.231**	0.294	0.242	0.303	0.307	0.295	0.274	0.285								
11. *Armina* sp. 6 (MW940327)	0.316	0.243	*0.223*	0.258	0.285	0.290	0.223	0.176	0.316	0.289							
12. *Armina* sp. 18 (MW940326)	0.335	0.279	0.259	0.269	0.291	0.272	0.310	0.269	0.264	0.296	0.250						
13. *Armina papillata* (MW940319)	0.361	0.295	0.311	0.282	0.292	0.284	0.332	0.300	0.002	0.285	0.316	0.264					
14. *Armina* sp. 8 (MW940306)	0.354	0.234	0.259	0.263	0.267	0.265	0.231	0.164	0.292	0.307	0.128	0.283	0.297				
15. *Armina* sp. B (MW940304)	0.252	0.240	0.263	0.233	0.272	0.273	0.300	0.249	0.247	0.260	0.254	0.243	0.247	0.288			
16. *Armina* sp. A (MW940303)	0.314	0.251	0.254	0.233	0.279	0.277	0.205	0.160	0.314	0.265	0.132	0.290	0.314	0.147	0.248		
17. *Armina* cf. *comta* (MW940302)	0.238	0.230	0.253	0.231	0.268	0.270	0.289	0.246	0.241	0.257	0.258	0.241	0.241	0.292	0.005	0.252	
