# Supplementary material for: ﻿Characterization of 11 complete mitochondrial genomes in Nudibranchia (Mollusca, Gastropoda)
Source: Zookeys. 2025 Jul 4;1244:61–86. doi: 10.3897/zookeys.1244.139617 (PMC12254827; doi:10.3897/zookeys.1244.139617)
Supplement: Supplementary material 2 — Supplementary figures S1–S4 [file zookeys-1244-061_article-139617__-s002.pdf]

Supplementary material 2

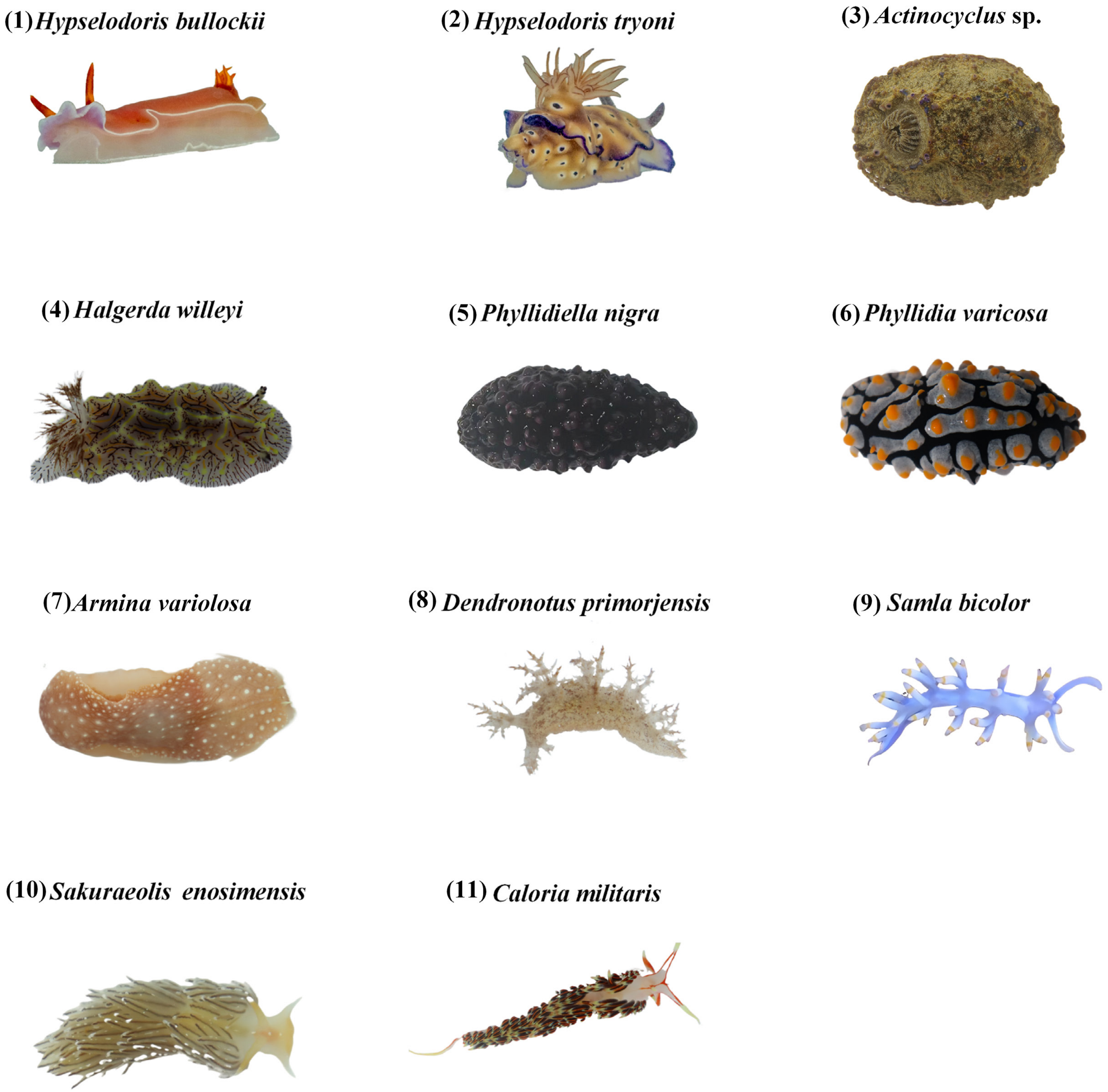

figure S1.

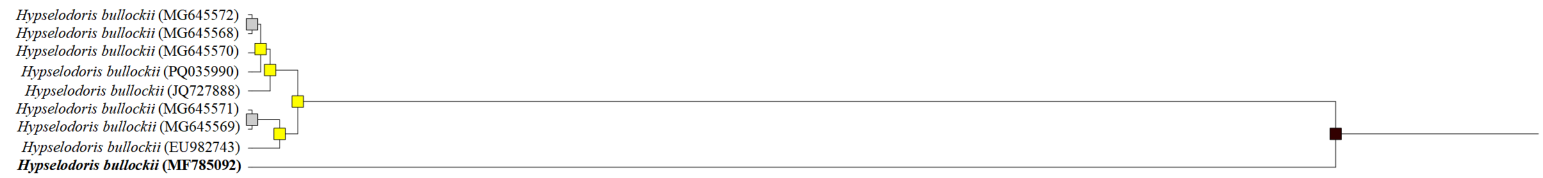

figure S2.

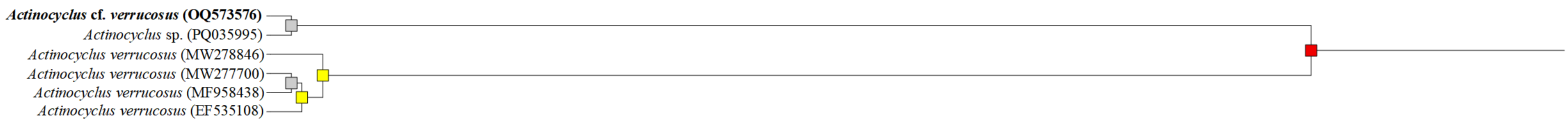

figure S3.

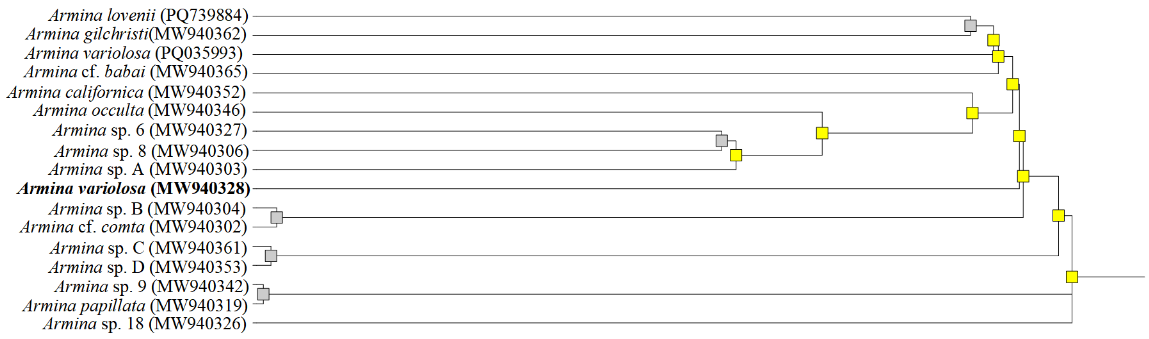

figure S4.
